# The subfunctionalization of *shox* and *shox2* paralogs in shark highlights both shared and distinct developmental mechanisms of branchial arches and fins

**DOI:** 10.3389/fcell.2025.1667637

**Published:** 2025-10-01

**Authors:** Galina V. Ermakova, Irina V. Meyntser, Vassily A. Lyubetsky, Andrey G. Zaraisky, Andrey V. Bayramov

**Affiliations:** ^1^ Shemyakin-Ovchinnikov Institute of Bioorganic Chemistry, Russian Academy of Sciences, Moscow, Russia; ^2^ Biological Department of the Mosqvarium Center for Oceanography and Marine Biology, Moscow, Russia; ^3^ Institute for Information Transmission Problems of the Russian Academy of Sciences (Kharkevich Institute), Moscow, Russia; ^4^ Koltzov Institute of Developmental Biology of Russian Academy of Sciences, Moscow, Russia; ^5^ Department of Regenerative Medicine of the Pirogov Russian National Research Medical University, Moscow, Russia

**Keywords:** *shox*, *shox2*, shark, median fins, paired fins, branchial arches

## Abstract

The genomes of most gnathostomes contain two paralogs of the *shox* gene, *shox* and *shox2*, both of which are implicated in the development of two key morphological innovations: the jaw apparatus derived from the branchial arches and the paired appendages, whose evolutionary origins remain debated. Here, we investigate the expression patterns of *shox* and *shox2* paralogs in the gray bamboo shark (*Chiloscyllium griseum*), a representative of Chondrichthyes, a basally divergent gnathostome lineage. The paired fins of cartilaginous fishes are considered a basal model for gnathostome appendages. Our findings suggest spatial subfunctionalization of the *shox* and *shox2* genes. Specifically, *shox* is expressed in the mandibular and branchial arches, as well as in paired and unpaired fins, indicating shared developmental mechanisms among these structures. In contrast, *shox2* expression is predominantly restricted to paired fins, highlighting distinct developmental features that differentiate them from the evolutionarily older median fins.

## Introduction

Accumulating evidence suggests that the evolution of body plans and the emergence of novel morphological structures are driven by genomic changes, encompassing both alterations in gene regulation and the gain or loss of specific genes (Rubinstein and de Souza, 2013; Long et al., 2016). For example, the emergence of the novel homeobox gene *Anf/Hesx1* in the ancestors of extant vertebrates established the prerequisites for the development of the telencephalon, a unique region of the vertebrate forebrain (Zaraisky et al., 1992; Kazanskaya et al., 1997; Ermakova et al., 2007; Bayramov et al., 2016). The loss of *actinodin* genes in the ancestors of tetrapods may have facilitated the evolution of limbs adapted for terrestrial locomotion (Zhang et al., 2010). Similarly, the loss of genes such as *c-Answer*, *Ag1*, *Ras-dva1*, and *Tfp4* in the ancestors of warm-blooded vertebrates has been proposed as one of the factors contributing to the reduced capacity for limb regeneration in these lineages, a capability that is retained in many extant cold-blooded species possessing these genes (Ivanova et al., 2013; 2015; 2018; Korotkova et al., 2019; Tereshina et al., 2019; Ivanova et al., 2021).

A more prevalent evolutionary mechanism than the emergence of entirely novel genes is the duplication of pre-existing ancestral genes, followed by functional divergence among the resulting paralogs (Ohno, 1970; Deem and Brisson, 2024). In the human genome, for instance, over 70% of genes possess at least one paralog (Ibn-Salem et al., 2017; Hu et al., 2022). Although the majority of paralogs are typically lost following duplication due to functional redundancy or degeneration, those that undergo subfunctionalization (partitioning of ancestral functions), neofunctionalization (acquisition of novel functions), or confer other selective advantages may be retained within the genome (Kuzmin et al., 2022; Rastogi and Liberles, 2005). The marked increase in morphological complexity observed in vertebrates, compared with their ancestral forms, is thought to result from two rounds of whole-genome duplication that took place during the early stages of vertebrate evolution (Ohno, 1970; Bayramov et al., 2021).

Vertebrates comprise two major evolutionary lineages, jawless (agnathans) and jawed (gnathostomes), which diverged during the Cambrian period, approximately 535–462 million years ago (Janvier, 2006; Kuraku and Kuratani, 2006; Feinberg and Mallatt, 2013; Bayramov et al., 2018). Jawless vertebrates originated in the Cambrian, flourished over the subsequent 150 million years during the Ordovician and Silurian, and experienced extinction of several major groups in the Devonian (Donoghue and Keating, 2014; Johanson, 2020). Extant jawless vertebrates are represented by lampreys and hagfish, comprising approximately 100 species (Shimeld and Donoghue, 2012; Kuraku, 2013). The earliest known jawed vertebrates are dated to the Early Silurian (Zhao and Zhu, 2010), and the Devonian period witnessed a significant diversification of gnathostomes, including the emergence of terrestrial forms by the Late Devonian (∼360 million years ago) (Brazeau and Friedman, 2015). Modern gnathostomes, encompassing over 50,000 species, include cartilaginous and bony fishes, amphibians, and terrestrial vertebrates (Kuraku, 2013; Brazeau and Friedman, 2015).

A defining feature of gnathostomes is the presence of both a jaw apparatus and paired appendages (Donoghue and Keating, 2014; Striedter and Northcutt, 2019). In contrast, extant jawless vertebrates lack paired appendages, and the homology between gnathostome limbs and the appendages of fossil jawless vertebrates remains contentious, primarily due to the limited paleontological evidence regarding the endoskeletal structure of fins in fossil taxa (Tanaka and Onimaru, 2012; Wilson et al., 2007; Bayramov et al., 2024). Consequently, paired appendages in gnathostomes are frequently considered evolutionary innovations, prompting investigations into the genetic mechanisms underlying their origin (Larouche et al., 2019). The bauplan of gnathostomes includes two paired appendage girdles, the pectoral and pelvic, which support the corresponding fins in fishes and limbs in tetrapods (Bayramov et al., 2024). Fishes also possess unpaired (median) fins, including dorsal (one or more), anal, and caudal fins, which provide stability and facilitate locomotion in the aquatic environment (Lauder et al., 2002).

Given their phylogenetic position as a basally divergent lineage of gnathostomes, cartilaginous fishes serve as a fundamental model for studying the paired appendages of vertebrates (Seixas et al., 2023; Thompson et al., 2021). The pectoral fins of sharks comprise endoskeletal elements (basalia and radials) as well as exoskeletal fin rays. The basal segment is formed by three elements, propterygium, mesopterygium, and metapterygium, which together constitute the tribasal fin architecture (Cass et al., 2021). The appendages of more derived lineages, such as bony fishes and tetrapods, have undergone extensive and divergent modifications, complicating direct comparisons between the developmental mechanisms of bony fish fins and tetrapod limbs, which are common laboratory models (Yano and Tamura, 2013; Hawkins et al., 2022; Thompson et al., 2021). This underscores the importance of studying the appendages of cartilaginous fishes, which represent the first appearance of paired appendages in vertebrate evolution and have largely retained their ancestral structural features (Cole and Currie, 2007).

Beyond the question of the origin of paired appendages in gnathostomes, considerable interest also surrounds the mechanisms driving the evolutionary transformation of ancestral fins into terrestrial limbs. Genetic studies suggest that this transformation may have involved changes in the expression of genes of *Hoxa* and *Hoxd* clusters (Woltering et al., 2020; Leite-Castro et al., 2016). It has been proposed that the capacity for these evolutionary innovations did not arise *de novo* in terrestrial vertebrates but was, at least in part, already present in their aquatic ancestors (Freitas et al., 2007).

The skeletal structure of tetrapod limbs comprises three proximodistally arranged segments: (1) the stylopod, a proximal segment with a single bone (the humerus in forelimbs or femur in hindlimbs), (2) the zeugopod, an intermediate segment consisting of two parallel bones (radius and ulna in the forelimb, tibia and fibula in the hindlimb), and (3) the autopod, a distal segment encompassing the mesopodium (wrist or ankle) and digits (Don et al., 2013).

The cranial skeleton, a hallmark of vertebrates, underwent considerable structural elaboration in gnathostomes, encompassing the development of both the jaw and the branchial apparatus (Fish, 2019). The formation of the craniofacial skeleton involves contributions from two cell types: neural crest cells (NCCs) and mesodermal cells (Kaucka and Adameyko, 2019; Hirasawa and Kuratani, 2015). The role of NCCs is particularly critical in the development of the anterior regions of the neurocranium and the branchial (also known as pharyngeal) arches (Kuratani, 2005). During early embryonic development, cranial NCCs (CNCCs) diversify into neural and non-neural (ectomesenchymal) lineages (Soldatov et al., 2019). Ectomesenchymal cells, representing an intermediate embryonic cell type, migrate to the branchial arches and contribute to the formation of various facial structures by differentiating into a variety of mesenchymal cell types, giving rise to bone, cartilage, teeth, and connective tissue (Feng et al., 2025). In the little skate (*Leucoraja erinacea*), developmental studies have demonstrated that the mandibular and hyoid arch skeletons are derived from neural crest-derived mesenchyme, the branchial arches arise from both neural crest- and mesoderm-derived skeletogenic mesenchyme, whereas the pectoral fin skeleton originates exclusively from mesoderm-derived mesenchyme (Sleight and Gillis, 2020).

Paralogs of the short stature homeobox genes, *shox* and *shox2*, have been identified as important regulators of paired appendage and craniofacial development in vertebrates (Supplementary Table 1; Abassah-Oppong et al., 2024; Decker et al., 2011; Blaschke et al., 2007; Espinoza-Lewis et al., 2009; Gu et al., 2008; Laureano et al., 2022; Rosin et al., 2015; Xu et al., 2019; Yu et al., 2005). In humans, *SHOX* plays a critical role in regulating longitudinal growth (Sabherwal et al., 2007). Mutations in *SHOX* are associated with several growth disorders and body disproportions, including Turner syndrome, Léri-Weill dyschondrosteosis (LWD), and Langer mesomelic dysplasia, which are notably characterized by the shortening of zeugopod elements in the limbs (Rao et al., 1997; Shears et al., 1998; Eduful, 2021; Sabherwal et al., 2007). The human *SHOX2* gene encodes a protein with 83% sequence homology to *SHOX* and an identical homeodomain (Blaschke et al., 1998; Yu et al., 2007). *Shox2* has been implicated in the development of the stylopod of both fore- and hindlimbs, craniofacial structures such as the temporomandibular joint and secondary palate, the facial motor nucleus and associated facial nerves, as well as neurons of the dorsal root ganglia (Abassah-Oppong et al., 2024; Cobb et al., 2006).

In human limb development, *SHOX* and *SHOX2* exhibit overlapping yet spatially distinct expression patterns, indicative of spatial subfunctionalization, with *SHOX2* expressed more proximally relative to *SHOX* (Clement-Jones et al., 2000; Yu et al., 2007). A similar spatial distinction is observed in the limbs of the axolotl, where *shox2* is expressed in a restricted proximal-posterior domain of the early limb bud, whereas *shox* is expressed more distally, occupying a broader region of the limb bud (Duerr et al., 2025). Notably, neither gene is expressed in the distal-most regions of the axolotl limb bud. In chicken embryos, *shox* expression is detected in the mesenchyme of the proximal two-thirds of the developing limb bud (Sabherwal et al., 2007).

In mice, the *Shox* gene is absent, and knockout of the remaining paralog *Shox2* results in defective development of the stylopod, the most proximal limb segment (Cobb et al., 2006; Yu et al., 2007). Expression of *shox* and *shox2* has also been documented in *Danio rerio* embryos; however, there remains insufficient data to allow a detailed comparison of the expression patterns of these two paralogs in *D. rerio* fin buds (Thisse and Thisse, 2004; Sawada et al., 2015). In addition to fin buds, *shox* expression in *D. rerio* has been reported in the olfactory pits, hatching gland, putative heart, branchial arches, and in CNCCs within the ventral-intermediate domains of the mandibular and hyoid arches (Kenyon et al., 2011; Askary et al., 2017). Morpholino knockdown of *D*. *rerio shox* impaired cell proliferation in the anterior region of pharyngula-stage embryos, which, in combination with data obtained from cultures of human mesenchymal stem cells (hMSCs), suggested that *shox/SHOX* maintains the population of embryonic bone progenitor cells by sustaining their proliferative state and repressing the onset of early osteogenic gene expression (Yokokura et al., 2017). Notably, in *D. rerio* fins, *shox2* has been identified as a downstream target of *shox* (Hoffmann et al., 2021).

The formation of endochondral skeletal elements in vertebrate limbs involves the sequential condensation of mesenchymal cells, the differentiation of cartilage, and its subsequent replacement by bone tissue (Long et al., 2016; Yu et al., 2007). In *Shox2* knockout mice, mesenchymal condensation occurs normally; however, the subsequent stages of bone development, chondrogenesis and ossification, are disrupted (Yu et al., 2007). Similar defects in endochondral ossification were observed in CRISPR-mediated *shox* knockouts in axolotl, although this effect was limited to the proximal limb segments, the stylopod and zeugopod, while ossification of the autopod elements proceeded normally (Duerr et al., 2025). This suggests that the regulatory mechanisms governing the skeletal elements of proximal versus distal limb segments in tetrapods differ, which is of particular interest in the context of the evolutionary transition from ancestral fins to terrestrial limbs and the development of the autopod, the homology of which with fin elements remains a matter of debate (Cass et al., 2021).

It has been demonstrated that *shox* and *shox2* genes are targets of retinoic acid (RA), a proximal signal involved in vertebrate limb development (Duerr et al., 2025; Feneck and Logan, 2020). It is hypothesized that *shox* expression is activated by the RA-dependent gene *meis1* and repressed by the distally expressed gene *hoxa13* (Duerr et al., 2025).

To investigate the roles of *shox* and *shox2* in basal vertebrate appendage and craniofacial development, we examined their expression patterns in embryos of the grey bamboo shark (*Chiloscyllium griseum*), a cartilaginous fish representing one of the basally divergent lineages of gnathostomes. To our knowledge, previous comparative, side-by-side analyses of *shox* and *shox2* expression have been limited to tetrapod species. Therefore, one of the aims of our study was to determine when the spatial subfunctionalization of *shox* paralogs arose during vertebrate evolution and to investigate its potential role as a contributing factor in the evolutionary transformation of ancestral fins into tetrapod limbs.

## Results

### *Shox* genes phylogeny

To investigate the phylogeny of *Shox* genes in gnathostomes, we performed a search for *shox* homologs in available databases and analyzed the phylogenetic relationships of their encoded protein sequences. In addition to representatives of various gnathostome classes, the analysis included lamprey (a representative jawless vertebrate), amphioxus and ascidians (representatives of invertebrate chordates), as well as species from hemichordates, cnidarians, and several protostome groups (including anthozoans, mollusks, and annelids).

**FIGURE 1:**
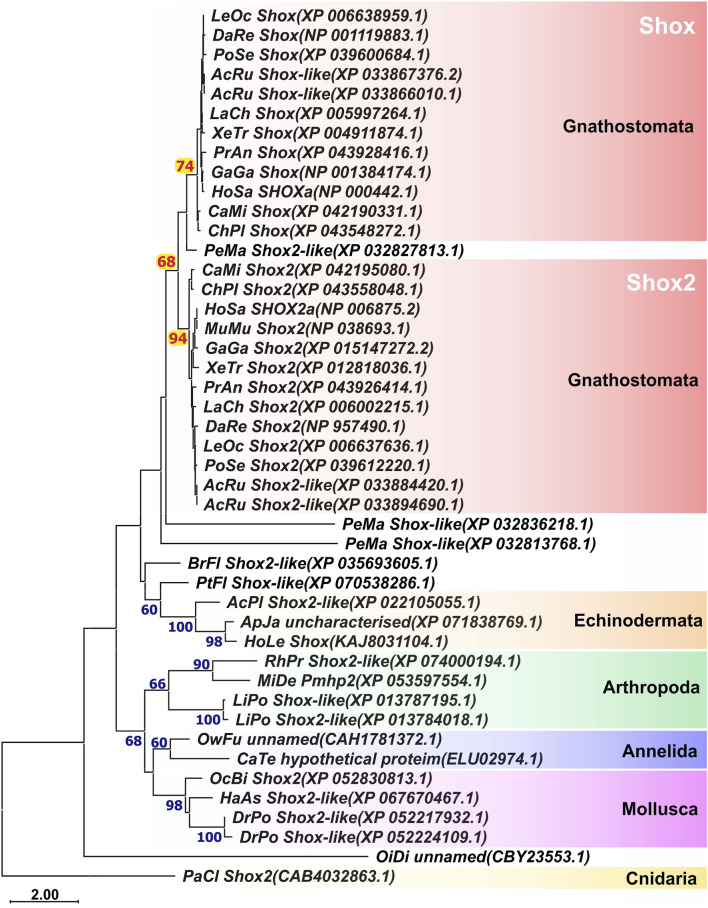
ML phylogenetic trees of Shox and Shox2 proteins. AcPl, *Acanthaster planci*; AcRu, *Acipenser ruthenus*; ApJa, Apostichopus japonicus; BrFl, *Branchiostoma floridae*; CaMi, *Callorhinchus milii*; CaTe, Capitella teleta; ChPl, Chiloscyllium plagiosum; DaRe, *Danio rerio*; DrPo, *Dreissena polymorpha*; GaGa, Gallus gallus; HaAs, Haliotis asinina; HoLe, Holothuria leucospilota; HoSa, *Homo sapiens*; LaCh, *Latimeria chalumnae*; LeOc–*Lepisosteus oculatus*; LiPo, Limulus polyphemus; MiDe, Microplitis demolitor; MuMu, *Mus musculus*; OcBi, Octopus bimaculoides; OiDi, *Oikopleura dioica*; OwFu, Owenia fusiformis; PaCl, Paramuricea clavata; PeMa, *Petromyzon marinus*; PoSe, Polypterus senegalus; PtFl, Ptychodera flava; RhPr, *Rhodnius prolixus*; XeTr, *Xenopus* tropicalis.

Phylogenetic analysis of *Shox* protein sequences (Figure 1) revealed that among chordates the stable presence of two *Shox* paralogs is unique to gnathostomes, with these paralogs being clearly distinguishable from one another (Supplementary Figure 1). In sterlet (*Acipenser ruthenus*), four *shox* paralogs were identified. In lampreys, three *shox* paralogs were identified. In the basal chordates, amphioxus and ascidian (*Oikopleura dioica*), only a single *shox* gene was present. Some analyzed invertebrate species possess two *shox* paralogs (e.g., *Limulus polyphemus* among arthropods and *Dreissena polymorpha* among mollusks); however, this feature is not consistently observed across other representatives within their respective groups.

### Expression of *shox* and *shox2* during the development of the grey bamboo shark *Chiloscyllium griseum*

Two paralogs, *shox* and *shox2*, were identified in the *C. griseum* genome. The expression patterns of these genes were analyzed in *C. griseum* embryos using *in situ* hybridization (ISH).

**FIGURE 2:**
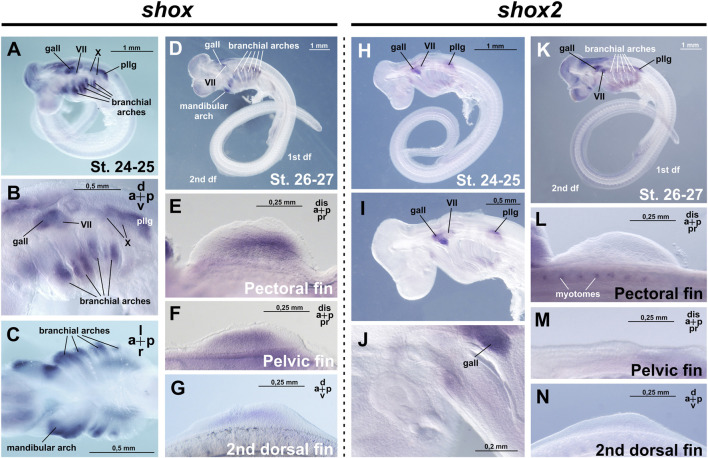
Expression of *shox* and *shox2* in *C. griseum* embryos at stages 24–27 (after Ballard et al., 1993). gall–ganglion of the anterior lateral line; VII–ganglion of the VII nerve; X–ganglion of the X nerve, pllg–posterior lateral line ganglion. At stages 24–26, *shox* expression is detected in the intermediate domains of the branchial arches **(A–D)** the mandibular arch, cranial ganglia, as well as in the paired and second dorsal fin buds **(E–G)**. At the same stages, *shox2* is expressed in the cranial and ganglia **(H–K)** and in the myotomes **(L)**. Notably, *shox2* expression is absent in the paired and second dorsal fins **(L–N)**. Expression patterns that were reproduced in at least 80% of cases were considered reliable.

The earliest stages examined were stages 24–25 (according to Ballard et al., 1993), when the primordia of paired fins are present as ectodermal thickenings but not yet externally visible, 1–5 pharyngeal clefts are open, and the ganglia of the anterior lateral line and cranial nerves can be detected. At these stages *shox* expression was detected in the intermediate domains of branchial arches, the visceral ganglia of cranial nerves VII and X, the ganglion of the anterior lateral line and posterior lateral line ganglion (Figures 2A–C).

By stage 27, when fin buds are already morphologically distinguishable, six pairs of pharyngeal clefts are open and external gill filaments appear on all branchial arches, additional *shox* expression domains appear in the paired (pectoral and pelvic) and unpaired dorsal fins, alongside persistent expression in the mandibular and branchial arches (Figures 2D–G).

**FIGURE 3:**
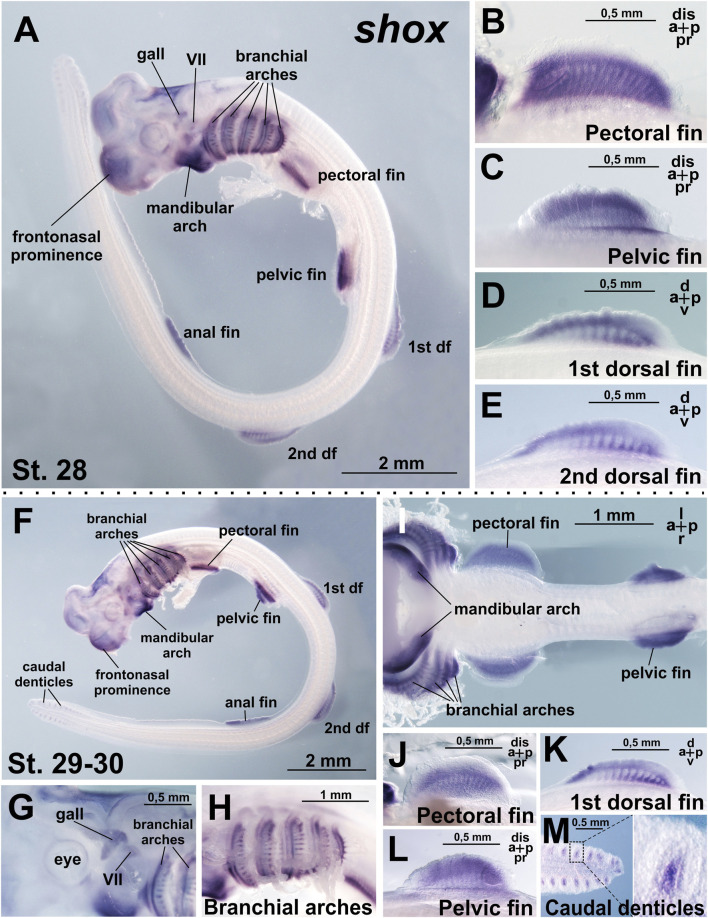
Expression of *shox* in *C. griseum* embryos at stages 28 **(A–E)** and 30 **(F–M)**. At stages 28–30 *shox* expression is observed in mandibular and branchial arches, including gill rays **(A,F,H,I)**, paired (pectoral and pelvic) **(A–C,F,I,J,L)** and median (dorsal and anal) **(A,D,E,F,K)** fins, cranial ganglia **(A,G)** frontonasal prominence **(A,F)** and caudal denticles **(M)**. df, dorsal fin; gall, ganglion of the anterior lateral line; VII, ganglion of the VII nerve. Expression patterns that were reproduced in at least 80% of cases were considered reliable.

At stage 28, when all paired (pectoral and pelvic) and median (dorsal and anal) fins are well developed, *shox* expression is detected in the pectoral, pelvic, both dorsal, and anal fins (Figures 3A–E). The expression pattern is highly specific to the fins, as the surrounding trunk tissues show no staining. Within the fin buds, expression is distributed across the entire structure but is not uniform, with areas corresponding to the primordia of endoskeletal elements staining less intensely than the surrounding tissues (Figures 3B–E). In the dorsal fins, expression heterogeneity is more pronounced, with the most intense staining localized to the antero-distal and postero-proximal regions. Beyond the fins, *shox* expression persists and expands in the mandibular arch, which is fully stained and within the branchial arches, where *shox* expression extends from the intermediate domain dorsally and ventrally and is observed in the gill rays (Figure 3A). *Shox* expression is also detected in the visceral ganglia, the ganglion of the anterior lateral line, and the frontonasal prominence, encompassing the dorsal telencephalon region and the area surrounding the nasal pits (Figure 3A).

The general pattern of *shox* expression remains consistent at stage 30 (Figures 3F–M).

In addition to fins, *shox* is expressed in the branchial arches and the proximal regions of the developing branchial rays (Figures 3F–I). *Shox* expression is particularly strong in the mandibular arch (Figures 3F,I). In the caudal fin, *shox* expression is detected in the caudal denticles (Figure 3M), which are transient structures formed by basal epithelium overlying the mesenchyme (Cooper et al., 2017). Embryonic caudal denticles are lost before or during hatching, when the general body denticles develop and occupy their positions. Denticles are structurally homologous to vertebrate teeth and perform multiple functions in adult sharks, including reducing hydrodynamic drag during locomotion, providing defensive armor, and facilitating communication via association with luminescent photophores (Cooper et al., 2023). *Shox* expression is localized to the mesenchymal core of the caudal denticles (Figure 3M).

At the earlier stages examined (stages 24–27 after Ballard et al., 1993), *shox2* expression is detected in the visceral ganglion of cranial nerve VII, the ganglion of the anterior lateral line, posterior lateral line ganglion and the myotomes, which are the precursors of the skeletal muscle of the body axis in vertebrates (Figures 2H–N; Hollway and Currie, 2003).

**FIGURE 4:**
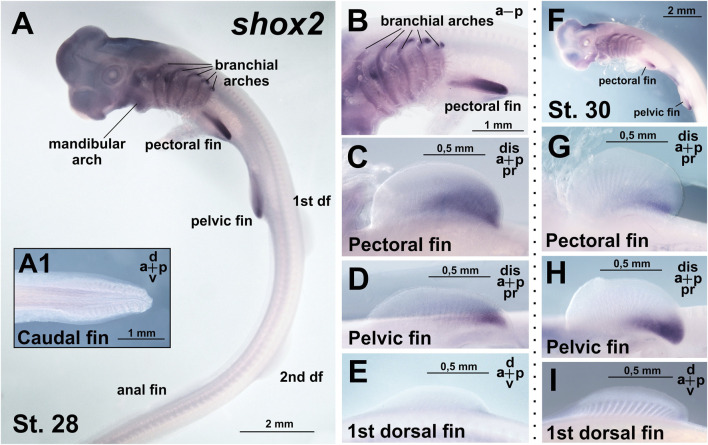
Expression of *shox2* in *C. griseum* embryos at stages 28 **(A–E)** and 30 **(F–I)**. At stages 28–30 *shox2* expression is observed in branchial arches **(A,B)** and posterior proximal domains of paired (pectoral and pelvic) fins **(A–D,F–H)**. *Shox2* expression was not detected in median (dorsal and anal) fins **(A,E,I)** and caudal denticles **(A1)**. df–dorsal fin. Expression patterns that were reproduced in at least 80% of cases were considered reliable.

From stages 28–30, *shox2* is expressed in the proximal-caudal region of the paired (pectoral and pelvic) fins (Figures 4A–H). In contrast, no *shox2* expression is detected in the unpaired (dorsal and anal) fins (Figures 4E,I) and caudal denticles (Figure 4A1). In the branchial arches, *shox2* is expressed as a thin medial stripe (Figures 4A,B,F). Expression is absent in the caudal fin and caudal denticles (Figure 4A1).

Comparison of *shox* and *shox2* expression in *C. griseum* embryos at stages 28–30 reveals shared expression in the paired fins (Figures 3, 4; Supplementary Figure 2) and, to some extent, in the branchial arches (Figures 3A,F–I; Figures 4A,B,F), although the expression patterns of the two paralogs differ in these structures. In the mandibular arch, *shox* expression is stronger and detected at earlier stages (Figures 2A,D), while *shox2* expression is first detected at stage 28 and only after prolonged staining (Figure 4A). Notably, only *shox* is expressed in the unpaired fins (Figures 3, 4; Supplementary Figure 2).

## Discussion

The phylogenetic analysis of *Shox* genes revealed that these genes are not unique to vertebrates but are present in the genomes of various phylogenetic groups, including both vertebrate and invertebrate taxa. This finding refines the earlier hypothesis by Clement-Jones et al. (2000), which suggested *Shox* genes as the new genes of vertebrates, linked to the evolution of skeletal structures.

The presence of multiple *Shox* paralogs in jawless vertebrates (three paralogs in the sea lamprey) and in jawed vertebrates (two paralogs in most groups), combined with the presence of a single *shox* paralog in basal chordates such as amphioxus and ascidians, supports the hypothesis that multiple *shox* paralogs arose via whole genome duplications during early vertebrate evolution (Ohno, 1970; Sacerdot et al., 2018; Simakov et al., 2020; Nakatani et al., 2021; Marlétaz et al., 2024; Yu et al., 2024). The presence of four *shox* paralogs in the sterlet (*Acipenser ruthenus*) likely reflects an additional whole genome duplication event that occurred within this lineage (Du et al., 2020; Redmond et al., 2023). The weak clustering of lamprey *Shox* proteins with those of jawed vertebrates may reflect the distinctive amino acid composition often described as the “lamprey dialect” (Onimaru and Kuraku, 2018).

The previously reported patterns of *shox* and *shox2* gene expression across representatives of different gnathostome classes are summarized in Supplementary Table 1. *Shox* and *shox2* paralogs are known to exhibit spatial subfunctionalization along the proximodistal axis of limb development (Clement-Jones et al., 2000; Yu et al., 2007). We also observed distinct expression patterns of *shox* and *shox2* in the paired fins of *C. griseum*. The *shox* is expressed broadly throughout the fin bud of both pectoral and pelvic fins, though not uniformly, whereas *shox2* expression is restricted to the proximal-posterior region of paired fins. In our experiments, we did not detect *shox2* expression in the unpaired fins (dorsal, anal, and caudal); however, the ISH data do not allow us to determine the absolute level of *shox2* expression, which may be greater than zero. However, considering that the ISH was performed on whole *C. griseum* embryos, the results indicate that *shox2* expression in unpaired fins is at least substantially lower than in paired fins, in contrast to *shox* gene expression. Such an observation in a representative of Chondrichthyes suggests that the regulatory mechanisms responsible for the spatial divergence of expression domains between the two *shox* paralogs may have been established early in the evolution of jawed vertebrates. The posterior expression of *shox2* coincides with the area of the future metapterygial basal element, which is thought to have given rise to the tetrapod limb (Cass et al., 2021). The proximal localization of *shox2* expression in the shark fin is similar to its expression in the axolotl limbs and the proximal limb (stylopod) in mammals (Duerr et al., 2025; Cobb et al., 2006). The broader expression of *shox* in shark fins relative to *shox2* parallels the expression of *shox* orthologs in axolotl (Duerr et al., 2025).

A notable feature of *shox* expression in the shark is its activity in the unpaired fins, including both dorsal fins and the anal fin. This observation supports the hypothesis that developmental mechanisms and regulatory elements first established in the evolutionarily older unpaired fins were subsequently co-opted during the emergence of paired fins (Freitas et al., 2006; Hawkins et al., 2022). However, the absence of *shox2* expression in the unpaired fins indicates that this co-option did not involve a wholesale duplication of developmental programs. Instead, the origin of paired fins appears to have required additional, lineage-specific mechanisms.

Taking this finding into account, it would be valuable in the future to compare, for example using ATAC-seq, the patterns of open chromatin in the vicinity of both genes in shark embryos. Combined with RNA-seq analyses of paired and unpaired fin buds, such data could help identify regulatory circuits that govern either shared or fin-type-specific expression. Comparable experiments could then be extended to the orthologs of shark *shox* expressed in the dorsal fin during lamprey development. Cross-species comparison of these datasets may ultimately reveal the archetypal regulatory circuit underlying gene expression in the unpaired fins of vertebrate ancestors.

In addition, it would also be promising to search for conserved non-coding elements (CNEs) in the vicinity of both genes by comparing their orthologs across several jawed vertebrate species, including sharks. Such elements may represent critical components of the regulatory circuits determining the spatiotemporal expression patterns of both genes. For *shox*, such an analysis identified 35 CNEs around this gene (Kenyon et al., 2011). Comparison of the sets of conserved elements between *shox* and *shox2* could therefore help pinpoint elements potentially important for expression in paired versus unpaired fins. Finally, to test the functional role of each of the two *shox* genes in paired fin development, it will be important to perform knockdown or knockout experiments in suitable experimental models, such as *D. rerio* or *Xenopus*. Notably, in *D. rerio* pectoral fins both genes are expressed in a manner similar to what we observed in shark: *shox* is expressed more broadly (Kenyon et al., 2011), whereas *shox2* is noted to be restricted to the AER and ventral part (Thisse, B. and Thisse, C., ZFIN Direct Data Submission ID: ZDB-PUB-040907-1, ZDB-GENE-040426-1457, ZDB-FIG-060216-773).

In addition to the fins, *shox* expression in *C. griseum* is detected in the mandibular and branchial arches from early developmental stages, and at later stages it extends to the proximal regions of the developing gill rays. At the earliest stages examined (stages 24–25), *shox* expression in the mandibular and branchial arches is confined to the intermediate domains, resembling the previously reported expression of *shox* in the ventral-intermediate domain of the mandibular and hyoid arches in *D. rerio* (Askary et al., 2017). As shown for the branchial arches of *D. rerio*, chondrogenesis begins in the intermediate domains and subsequently extends into the ventral and dorsal domains (Barske et al., 2016).

Gill rays, characteristic of the branchial apparatus in chondrichthyans, develop on the posterior surface of the hyoid and gill arches in elasmobranchs (Gillis et al., 2009). A distal signaling center, the gill arch epithelial ridge (GAER), plays a key role in their development, serving as a source of sonic hedgehog (Shh) signaling (Gillis et al., 2011; Gillis and Hall, 2016; Rees et al., 2023). The presence of gill rays in cartilaginous fishes contributed to Gegenbaur’s hypothesis that paired appendages originated from the posterior branchial arch (Gegenbaur, 1878; Gillis and Hall, 2016). Subsequent studies have revealed shared features of gene expression and regulatory activity between gill arches and fins, involving key signaling pathways such as *Shh*, *Fgf*, *Wnt*, and RA (Akimenko et al., 1994; Gillis et al., 2009; Gillis et al., 2011; Gillis and Hall, 2016; Rees et al., 2023). Additionally, it has been demonstrated that, alongside NCCs, lateral plate mesoderm-derived cells, essential for paired fin development, also contribute to gill arch development (Sleight and Gillis, 2020; Prummel et al., 2020). The *shox* expression we observed in the gill arches, including the developing gill rays, and in paired fins of the shark further supports the developmental similarities between these structures.

The expression of *shox* in the mandibular arch reflects its ontogenetic similarity to branchial arches (Gillis et al., 2013). *Shox* expression in the primordia of paired fins and branchial arches may indicate an evolutionary relationship between these structures, tracing back to the ideas of Gegenbaur (Gillis and Hall, 2016). However, *shox* expression is also detected in a range of other structures, such as the mandibular arch and median fins. A comparable spectrum of expression domains, including the caudal denticles, was observed for the shark ortholog of the novel gnathostome gene *chordin-like1* (Ermakova et al., 2025). Although the expression patterns of *shox* and *chordin-like1* differ in detail, such distributional similarities may point to shared underlying regulatory mechanisms governing the formation of these structures in gnathostomes.

Although the tissue sources of anterior craniofacial structures, the visceral arches, and fin/limb buds differ, their developmental cellular mechanisms share some common features. The formation of all these structures involves populations of mesenchymal cells arising through epithelial-to-mesenchymal transition (EMT) (Theveneau and Mayor, 2012; Gros and Tabin, 2014). In the case of the anterior craniofacial structures and the mandibular arch, these cells consist of neural crest–derived ectomesenchyme; branchial arches are formed by a combination of neural crest–derived ectomesenchyme and mesenchyme derived from the paraxial and anterior lateral plate mesoderm; and the buds of paired appendages develop from lateral plate mesoderm–derived mesenchyme (Feng et al., 2025; Sleight and Gillis, 2020; Kaucka et al., 2016). Moreover, similar regulatory signals, including Shh, FGFs, and BMPs, play critical instructive roles in both craniofacial and appendage development (Kaucka et al., 2016). Within this context, the expression of *shox* in cranial structures, the mandibular and branchial arches, and fin buds may reflect “deep homology” (after Shubin et al., 2009) of the underlying mechanisms governing craniofacial and appendage development in vertebrates.

Similarly, the development of paired and unpaired fins exhibits comparable regulatory signals, suggesting that the origin of paired appendages involved the redeployment of genetic programs from the paraxial to the lateral mesoderm (Freitas et al., 2006; Hawkins et al., 2022). The observed expression of *shox* in both unpaired and paired fins of sharks is consistent with this hypothesis.

In mammals, including mice and humans, the tissue-specific activity of *Shox* and *Shox2* is regulated by cis-regulatory elements, notably enhancers located within extensive gene deserts downstream (centromeric) of these transcription factor genes (Abassah-Oppong et al., 2024; Rosin et al., 2013). Comparable extended regulatory landscapes have been described for other key genes involved in signaling center formation during paired appendage development, such as *Shh* and *Fgf8* (Symmons et al., 2016; Marinić et al., 2013). It is plausible that such regulatory regions, enabling the expression of *shox* genes in multiple embryonic structures, including the mandibular and gill arches, paired and unpaired fins, and cranial nerve ganglia as observed in the shark, originated early in the evolution of jawed vertebrates and were inherited by more derived lineages.

## Materials and methods

### Animals and samples preparation

The animal study protocol was approved by the Institutional Review Board (or Ethics Committee) of the Shemyakin-Ovchinnikov Institute of Bioorganic Chemistry (Moscow, Russia, protocol code IACUC 229 dated 1 February 2018). The study was conducted in accordance with the local legislation and institutional requirements.

*Chiloscyllium griseum* eggs and embryos were collected in collaboration with the scientific department of the Moskvarium Center for Oceanography and Marine Biology (Moscow, Russia). The embryos of *C. griseum* were staged in accordance with Ballard et al., 1993. The choice of *C. griseum* as a representative of Chondrichthyes was motivated by the technical availability of embryos of this species in sufficient quantities.

For ISH, embryos were fixed in MEMFA solution (3.7% formaldehyde, 100 mM MOPS, 2 mM EGTA, 1 mM MgSO4), dehydrated in methanol and kept at −20 °C.

*Chiloscyllium griseum* total RNA sample was obtained from lysed embryos (3 embryos for probe) by purification with the Analytic Jena innuPREP RNA Mini Kit 2.0 (Berlin, Germany).

### Phylogenetic analysis

Sequence similarity searches of Shox and Shox2 homologs were conducted using the NCBI BLAST web server (NCBI Resource Coordinators, 2025) and the BLAST + suite (Camacho et al., 2009). The original BLAST algorithm was described by Altschul et al. (1990).

Translated nucleotide searches were performed using the TBLASTN algorithm (Gertz et al., 2006) via the NCBI BLAST web server (NCBI Resource Coordinators, 2025).

We checked available Nucleotide collections (nr/nt) and whole genome shotgun contigs (wgs) databases.

Multiple sequence alignment was performed using ClustalW in MEGA11 software (Gertz et al., 2006).

Phylogenetic analyses of Shox and Shox2 protein sequences of vertebrates were performed via the Maximum Likehood (ML) methods using the MEGA11 program (Tamura et al., 2021).

The choosing of optimal model was performed in MEGA11.

In ML method JTT matrix-based model (Jones et al., 1992) with Gamma distribution was used. The percentage of trees in which the associated taxa clustered together in the bootstrap test (500 replicates) is shown next to the branches (Felsenstein, 1985). The tree is drawn to scale, with branch lengths measured in the number of substitutions per site. The analysis involved 45 amino acid sequences.

The list of the analyzed Shox and Shox2 sequences is attached in Supplementary Information.

### *Shox* and *shox2* cDNA obtaining, ISH

*Chiloscyllium griseum shox and shox2* cDNAs were obtained by PCR with following primers:

ChGr_shox_full_Frw1; CAGCGAGCGGGCGAGCTAAC;

ChGr_shox_full_Rev1; CCCCGGCCCGGCTGATTG;

ChGr_shox_full_Frw2; ATTAGATCTGCCACCATGGAGGAGCTAACGGCGTT;

ChGr_shox_full_Rev2; AATGTCGACTCAGAGGCCCAGCGCCTCGG;

ChGr_shox2_full_Frw1; GGGACATATTCCTCCGAACA;

ChGr_shox2_full_Rev1; GATTTGGAATCACTGTTCGG;

ChGr_shox2_full_Frw2; ATTAGATCTGCCACCATGGAAGAACTTACAGCTTT;

ChGr_shox2_full_Rev2; AATCTCGAGTCACAGCCCTAGTGCTGCAG.

Nested PCR (Frw1/Rev1 => Frw2/Rev2) was performed with Encyclo polymerase Evrogen kit (www.evrogen.ru, Moscow).

The resulting cDNA fragments were cloned into the pAL2-T vector (Evrogen, Moscow) and cDNA inserts of three clones of each gene were sequenced.

ISH was carried out according to the protocol described by Ermakova et al. (2024).

The probe concentration was 2 μg/mL. Prior to hybridization, shark embryos were incubated in a solution containing 0.4% hydrogen peroxide, 0.5× SSC, and 5% formamide for 30 min at room temperature under a fluorescent lamp with constant agitation.

Pre-hybridization and hybridization were performed at 57 °C. The pre-hybridization step lasted 3 h, followed by hybridization for 16 h.

Post-hybridization washes were carried out for 2 h at 57 °C using hybridization buffer (twice for 30 min), followed by hybridization buffer mixed 1:1 with (2× SSC +0.1% Tween-20) (twice for 30 min). Subsequent washes were performed at 30 °C in (2× SSC +0.1% Tween-20) (five times for 10 min), and in (0.2× SSC +0.1% Tween-20) (twice for 20 min).

For the analysis of *shox* and *shox2* expression patterns in ISH, at least 5 *C. griseum* embryos from each of the presented stages were analysed. Expression patterns that were reproduced in at least 80% of cases were considered reliable.

Photography was carried out using a Leica M205 stereo microscope.

## Data Availability

The original contributions presented in the study are included in the article/Supplementary Material, further inquiries can be directed to the corresponding author.

## Supplementary material

SUPPLEMENTARY FIGURE 1 ML phylogenetic trees of Shox and Shox2 proteins of gnathostomes. Bootstraps >50 are shown. The Shox and Shox2 proteins of gnathostomes clearly segregate into two distinct clusters.

SUPPLEMENTARY FIGURE 2 Comparative expression of shox and shox2 in C. griseum embryos at stages 28 – 30 (after Ballard et al., 1993). In contrast to shox2, the shox gene is expressed in the mandibular arch and the median (dorsal) fins. Moreover, the domain of shox expression in the paired fins and branchial arches is broader than that of shox2. Expression patterns that were reproduced in at least 80% of cases were considered reliable.

SUPPLEMENTARY TABLE 1 Comparison of reported shox and shox2 genes expression across representatives of different gnathostome classes.

## Appendix

### Branchial (or pharyngeal, or gill) arches

Paired cartilaginous or bony loops located posterior to the pharynx that support the gills. The first branchial arch, the mandibular arch, gives rise to the jaws in jawed fishes.

### Caudal denticles

Transient structures formed by basal epithelium overlying the mesenchyme. Embryonic caudal denticles are lost before or during hatching, when the general body denticles develop and occupy their positions. Denticles are structurally homologous to vertebrate teeth and perform multiple functions in adult sharks, including reducing hydrodynamic drag during locomotion, providing defensive armor, and facilitating communication via association with luminescent photophores.

### Orthologs

Homologous genes or proteins in different organisms (species) that arose through speciation.

### Paired appendages

Bilateral structures located along the sides of the body that function in active locomotion, including anterior appendages (pectoral fins in fish) and posterior appendages (pelvic fins in fish). In the paleontological record, paired fins appear later than median (unpaired) fins. Two main hypotheses have been proposed for their evolutionary origin, the Balfour–Thacher–Mivart lateral fold theory and Gegenbaur’s branchial arch theory.

### Paralogs

Homologous genes within a single organism (species) that originated through duplication of an ancestral gene.

### Pharyngeal clefts (or branchial clefts)

A series of ectoderm-derived external grooves or slits that give rise to openings on the lateral surfaces of the pharynx. These structures facilitate the passage of water from the oral cavity to the external environment, thereby enabling the irrigation of gills and supporting respiratory function.

### Propterygium, mesopterygium, and metapterygium

Anterior-to-posteriorly arranged basal (proximal) endoskeletal elements of paired fins that connect the fin blade to the girdle. A complete set of these elements is found in the fins of cartilaginous fishes, whereas in bony fishes the metapterygium is reduced. In contrast, the tetrapod limb is considered to be derived from the metapterygium.

### Stylopod, zeugopod, and autopod

The proximal-to-distal skeletal elements of the tetrapod limb. The stylopod and zeugopod are homologous to elements of the ancestral metapterygial axis, whereas the origin of the autopod remains debated, with hypotheses including the digital arch theory and the Turing self-organization mechanism.

### Unpaired (or median) fins

Fins located along the midline of the fish body, including the dorsal fins (two in sharks), the anal fin, and the caudal fin. Median fins play a crucial role in stability, maneuverability, and forward propulsion during swimming. They are considered an evolutionarily ancient type of fin, and their developmental genetic program was later co-opted during the emergence of paired fins. Unlike paired fins, median fins are also present in extant jawless vertebrates.

## References

[B1] Abassah-Oppong S. Zoia M. Mannion B. J. Rouco R. Tissières V. Spurrell C. H. (2024). A gene desert required for regulatory control of pleiotropic Shox2 expression and embryonic survival. Nat. Commun. 15 (1), 8793. 10.1038/s41467-024-53009-7 39389973 PMC11467299

[B2] Akimenko M. A. Ekker M. Wegner J. Lin W. Westerfield M. (1994). Combinatorial expression of three zebrafish genes related to distal-less: part of a homeobox gene code for the head. J. Neurosci. 14 (6), 3475–3486. 10.1523/JNEUROSCI.14-06-03475.1994 7911517 PMC6576961

[B3] Altschul S. F. Gish W. Miller W. Myers E. W. Lipman D. J. (1990). Basic local alignment search tool. J. Mol. Biol. 215, 403–410. 10.1016/S0022-2836(05)80360-2 2231712

[B4] Askary A. Xu P. Barske L. Bay M. Bump P. Balczerski B. (2017). Genome-wide analysis of facial skeletal regionalization in zebrafish. Development 144, 2994–3005. 10.1242/dev.151712 28705894 PMC5592815

[B5] Ballard W. W. Mellinger J. Lechenault H. (1993). A series of normal stages for development of *Scyliorhinus canicula*, the lesser spotted dogfish (*chondrichthyes: scyliorhinidae*). J. Exp. Zoology 267 (3), 318–336. 10.1002/jez.1402670309

[B6] Barske L. Askary A. Zuniga E. Balczerski B. Bump P. Nichols J. T. (2016). Competition between jagged-notch and Endothelin1 signaling selectively restricts cartilage formation in the zebrafish upper face. PLoS Genet. 12, e1005967. 10.1371/journal.pgen.1005967 27058748 PMC4825933

[B7] Bayramov A. V. Ermakova G. V. Eroshkin F. M. Kucheryavyy A. V. Martynova N. Y. Zaraisky A. G. (2016). The presence of Anf/Hesx1 homeobox gene in lampreys suggests that it could play an important role in emergence of telencephalon. Sci. Rep. 6, 39849. 10.1038/srep39849 28008996 PMC5180219

[B8] Bayramov A. V. Ermakova G. V. Kucheryavyy A. V. Zaraisky A. G. (2018). Lampreys, “Living Fossils,” in research on early development and regeneration in vertebrates. Russ. J. Dev. Biol. 49 (6), 327–338. 10.1134/S1062360418080015

[B9] Bayramov A. V. Ermakova G. V. Kuchryavyy A. V. Zaraisky A. G. (2021). Genome duplications as the basis of vertebrates’ evolutionary success. Russ. J. Dev. Biol. 52, 141–163. 10.1134/S1062360421030024

[B10] Bayramov A. V. Yastrebov S. A. Mednikov D. N. Araslanova K. R. Ermakova G. V. Zaraisky A. G. (2024). Paired fins in vertebrate evolution and ontogeny. Evol. Dev. 26, e12478. 10.1111/ede.12478 38650470

[B11] Blaschke R. J. Monaghan A. P. Schiller S. Schechinger B. Rao E. Padilla-Nash H. (1998). SHOT, a SHOX-Related homeobox gene, is implicated in craniofacial, brain, heart, and limb development. Proc. Natl. Acad. Sci. U. S. A. 95, 2406–2411. 10.1073/pnas.95.5.2406 9482898 PMC19357

[B12] Blaschke R. J. Hahurij N. D. Kuijper S. Just S. Wisse L. J. Deissler K. (2007). Targeted mutation reveals essential functions of the homeodomain transcription factor Shox2 in sinoatrial and pacemaking development. Circulation 115, 1830–1838. 10.1161/CIRCULATIONAHA.106.637819 17372176

[B13] Brazeau M. D. Friedman M. (2015). The origin and early phylogenetic history of jawed vertebrates. Nature 520 (7548), 490–497. 10.1038/nature14438 25903631 PMC4648279

[B14] Camacho C. Coulouris G. Avagyan V. Ma N. Papadopoulos J. Bealer K. (2009). BLAST+: architecture and applications. BMC Bioinforma. 10, 421. 10.1186/1471-2105-10-421 20003500 PMC2803857

[B15] Cass A. N. Elias A. Fudala M. L. Knick B. D. Davis M. C. (2021). Conserved mechanisms, novel anatomies: the developmental basis of fin evolution and the origin of limbs. Diversity 13 (8), 384. 10.3390/d13080384

[B16] Clement-Jones M. Schiller S. Rao E. Blaschke R. J. Zuniga A. Zeller R. (2000). The short stature homeobox gene SHOX is involved in skeletal abnormalities in Turner syndrome. Hum. Mol. Genet. 9 (5), 695–702. 10.1093/hmg/9.5.695 10749976

[B17] Cobb J. Dierich A. Huss-Garcia Y. Duboule D. (2006). A mouse model for human short-stature syndromes identifies Shox2 as an upstream regulator of Runx2 during long-bone development. Proc. Natl. Acad. Sci. U. S. A. 103 (12), 4511–4515. 10.1073/pnas.0510544103 16537395 PMC1450202

[B18] Cole N. J. Currie P. D. (2007). Insights from sharks: evolutionary and developmental models of fin development. Dev. Dyn. 236 (9), 2421–2431. 10.1002/dvdy.21268 17676641

[B19] Cooper R. L. Martin K. J. Rasch L. J. Fraser G. J. (2017). Developing an ancient epithelial appendage: FGF signalling regulates early tail denticle formation in sharks. EvoDevo 8, 8. 10.1186/s13227-017-0071-0 28469835 PMC5414203

[B20] Cooper R. L. Nicklin E. F. Rasch L. J. Fraser G. J. (2023). Teeth outside the mouth: the evolution and development of shark denticles. Evol. and Dev. 25, 54–72. 10.1111/ede.12427 36594351

[B21] Decker E. Durand C. Bender S. Rödelsperger C. Glaser A. Hecht J. (2011). FGFR3 is a target of the homeobox transcription factor SHOX in limb development. Hum. Mol. Genet. 20 (8), 1524–1535. 10.1093/hmg/ddr030 21273290

[B22] Deem K. D. Brisson J. A. (2024). Problems with paralogs: the promise and challenges of gene duplicates in evo-devo research. Integr. Comp. Biol. 64 (2), 556–564. 10.1093/icb/icae009 38565319 PMC11406157

[B23] Don E. K. Currie P. D. Cole N. J. (2013). The evolutionary history of the development of the pelvic fin/hindlimb. Front. Cell Dev. Biol. 222 (1), 114–133. 10.1111/j.1469-7580.2012.01557.x 22913749 PMC3552419

[B24] Donoghue P. C. J. Keating J. N. (2014). Early vertebrate evolution. Palaeontology 57, 879–893. 10.1111/pala.12125

[B25] Du K. Stöck M. Kneitz S. Klopp C. Woltering J. M. Adolfi M. C. (2020). The sterlet sturgeon genome sequence and the mechanisms of segmental rediploidization. Nat. Ecol. Evol. 4 (6), 841–852. 10.1038/s41559-020-1166-x 32231327 PMC7269910

[B26] Duerr T. J. Miller M. Kumar S. Bakr D. Griffiths J. R. Gautham A. K. (2025). Retinoic acid breakdown is required for proximodistal positional identity during axolotl limb regeneration. Nat. Commun. 16 (1), 4798. 10.1038/s41467-025-59497-5 40494878 PMC12152164

[B27] Eduful J. (2021). SHOX and SHOX2 share a tissue-specific functional redundancy in temporomandibular joint formation. Sci. J. Biol. and Life Sci. 2 (1), 528. 10.33552/SJBLS.2021.02.000528

[B28] Ermakova G. V. Solovieva E. A. Martynova N. Y. Zaraisky A. G. (2007). The homeodomain factor xanf represses expression of genes in the presumptive rostral forebrain that specify more caudal brain regions. Dev. Biol. 307, 483–497. 10.1016/j.ydbio.2007.03.524 17511981

[B29] Ermakova G. V. Meyntser I. V. Zaraisky A. G. Bayramov A. V. (2024). Adaptation of the *in situ* hybridization method for working with embryos and larvae of modern representatives of phylogenetically ancient groups of vertebrates: lampreys, cartilaginous fishes and sturgeons. Russ. J. Dev. Biol. 55, 284–295. 10.1134/S1062360424700255

[B30] Ermakova G. V. Meyntser I. V. Mugue N. S. Lyubetsky V. A. Zaraisky A. G. Bayramov A. V. (2025). The emergence of chordin-like1 in gnathostomes May have contributed to the evolution of paired appendages. Front. Cell Dev. Biol. 13, 1649996. 10.3389/fcell.2025.1649996 40950401 PMC12425909

[B31] Espinoza-Lewis R. A. Yu L. He F. Liu H. Tang R. Shi J. (2009). Shox2 is essential for the differentiation of cardiac pacemaker cells by repressing Nkx2-5. Dev. Biol. 327, 376–385. 10.1016/j.ydbio.2008.12.028 19166829 PMC2694185

[B32] Feinberg T. E. Mallatt J. (2013). The evolutionary and genetic origins of consciousness in the Cambrian period over 500 million years ago. Front. Psychol. 4, 667. 10.3389/fpsyg.2013.00667 24109460 PMC3790330

[B33] Felsenstein J. (1985). Confidence limits on phylogenies: an approach using the bootstrap. Evolution 39, 783–791. 10.1111/j.1558-5646.1985.tb00420.x 28561359

[B34] Feneck E. Logan M. (2020). The role of retinoic acid in establishing the early limb bud. Biomolecules 10 (2), 312. 10.3390/biom10020312 32079177 PMC7072211

[B35] Feng J. Janečková E. Guo T. Ziaei H. Zhang M. Geng J. J. (2025). High-resolution spatial transcriptomics and cell lineage analysis reveal spatiotemporal cell fate determination during craniofacial development. Nat. Commun. 16, 4396. 10.1038/s41467-025-59206-2 40355462 PMC12069723

[B36] Fish J. L. (2019). Evolvability of the vertebrate craniofacial skeleton. Front. Cell Dev. Biol. 91, 13–22. 10.1016/j.semcdb.2017.12.004 29248471 PMC5999547

[B37] Freitas R. Zhang G. Cohn M. J. (2006). Evidence that mechanisms of fin development evolved in the midline of early vertebrates. Nature 442 (7106), 1033–1037. 10.1038/nature04984 16878142

[B38] Freitas R. Zhang G. Cohn M. J. (2007). Biphasic hoxd gene expression in shark paired fins reveals an ancient origin of the distal limb domain. PLoS ONE 2 (8), e754. 10.1371/journal.pone.0000754 17710153 PMC1937022

[B39] Gegenbaur C. (1878). “Grundzüge der vergleichenden Anatomie,” in Wilhelm engelmann.

[B40] Gertz E. Yu Y.-K. Agarwala R. Schäffer A. A. Altschul S. F. (2006). Composition-based statistics and translated nucleotide searches: improving the TBLASTN module of BLAST. BMC Biol. 4, 41. 10.1186/1741-7007-4-41 17156431 PMC1779365

[B41] Gillis J. A. Hall B. K. (2016). A shared role for sonic hedgehog signalling in patterning chondrichthyan gill arch appendages and tetrapod limbs. Development 143 (8), 1313–1317. 10.1242/dev.133884 27095494

[B42] Gillis J. A. Dahn R. D. Shubin N. H. (2009). Chondrogenesis and homology of the visceral skeleton in the little skate, *Leucoraja Erinacea* (*chondrichthyes: batoidea*). J. Morphol. 270 (5), 628–643. 10.1002/jmor.10710 19117064

[B43] Gillis J. A. Rawlinson K. A. Bell J. Lyon W. S. Baker C. V. Shubin N. H. (2011). Holocephalan embryos provide evidence for gill arch appendage reduction and opercular evolution in cartilaginous fishes. Proc. Natl. Acad. Sci. U. S. A. 108 (4), 1507–1512. 10.1073/pnas.1012968108 21220324 PMC3029744

[B44] Gillis J. Modrell M. Baker C. (2013). Developmental evidence for serial homology of the vertebrate jaw and gill arch skeleton. Nat. Commun. 4, 1436. 10.1038/ncomms2429 23385581 PMC3600657

[B45] Gros J. Tabin C. J. (2014). Vertebrate limb bud formation is initiated by localized epithelial-to-mesenchymal transition. Science 343, 1253–1256. 10.1126/science.1248228 24626928 PMC4097009

[B46] Gu S. Wei N. Yu L. Fei J. Chen Y. (2008). Shox2-deficiency leads to dysplasia and ankylosis of the temporomandibular joint in mice. Mech. Dev. 125, 729–742. 10.1016/j.mod.2008.04.003 18514492 PMC3010750

[B47] Hawkins M. B. Jandzik D. Tulenko F. J. Cass A. N. Nakamura T. Shubin N. H. (2022). An fgf-shh positive feedback loop drives growth in developing unpaired fins. Proc. Natl. Acad. Sci. U. S. A. 119 (10), e2120150119. 10.1073/pnas.2120150119 35238632 PMC8916008

[B48] Hirasawa T. Kuratani S. (2015). Evolution of the vertebrate skeleton: morphology, embryology, and development. Zool. Lett. 1, 2. 10.1186/s40851-014-0007-7 26605047 PMC4604106

[B49] Hoffmann S. Roeth R. Diebold S. Gogel J. Hassel D. Just S. (2021). Identification and tissue-specific characterization of novel SHOX-Regulated genes in zebrafish highlights SOX family members among other genes. Front. Genet. 12, 688808. 10.3389/fgene.2021.688808 34122528 PMC8191631

[B50] Hollway G. E. Currie P. D. (2003). Myotome meanderings. Cellular morphogenesis and the making of muscle. EMBO Rep. 4, 855–860. 10.1038/sj.embor.embor920 12949585 PMC1326358

[B51] Hu Y. Ewen-Campen B. Comjean A. Rodiger J. Mohr S. E. Perrimon N. (2022). Paralog explorer: a resource for mining information about paralogs in common research organisms. Comput. Struct. Biotechnol. J. 20, 6570–6577. 10.1016/j.csbj.2022.11.041 36467589 PMC9712503

[B52] Ibn-Salem J. Muro E. M. Andrade-Navarro M. A. (2017). Co-regulation of paralog genes in the three-dimensional chromatin architecture. Nucleic Acids Res. 45 (1), 81–91. 10.1093/nar/gkw813 27634932 PMC5224500

[B53] Ivanova A. S. Tereshina M. B. Ermakova G. V. Belousov V. V. Zaraisky A. G. (2013). Agr genes, missing in amniotes, are involved in the body appendages regeneration in frog tadpoles. Sci. Rep. 3, 1279. 10.1038/srep01279 23412115 PMC3573343

[B54] Ivanova A. S. Shandarin I. N. Ermakova G. V. Minin A. A. Tereshina M. B. Zaraisky A. G. (2015). The secreted factor Ag1 missing in higher vertebrates regulates fins regeneration in *Danio rerio* . Sci. Rep. 29 (5), 8123. 10.1038/srep08123 25630240 PMC4309956

[B55] Ivanova A. S. Korotkova D. D. Ermakova G. V. Martynova N. Y. Zaraisky A. G. Tereshina M. B. (2018). Ras-dva small GTPases lost during evolution of amniotes regulate regeneration in anamniotes. Sci. Rep. 8 (1), 13035. 10.1038/s41598-018-30811-0 30158598 PMC6115384

[B56] Ivanova A. S. Tereshina M. B. Araslanova K. R. Martynova N. Y. Zaraisky A. G. (2021). The secreted protein disulfide isomerase Ag1 lost by ancestors of poorly regenerating vertebrates is required for *Xenopus laevis* tail regeneration. Front. Cell Dev. Biol. 9, 738940. 10.3389/fcell.2021.738940 34676214 PMC8523854

[B57] Janvier P. (2006). Palaeontology: modern look for ancient lamprey. Nature 443 (7114), 921–924. 10.1038/443921a 17066021

[B58] Johanson Z. (2020). Vertebrate evolution: jawless heads go with the flow. Curr. Biol. 30 (23), R1431-R1433–R1433. 10.1016/j.cub.2020.09.055 33290712

[B59] Jones D. T. Taylor W. R. Thornton J. M. (1992). The rapid generation of mutation data matrices from protein sequences. Comput. Appl. Biosci. 8, 275–282. 10.1093/bioinformatics/8.3.275 1633570

[B60] Kaucka M. Adameyko I. (2019). Evolution and development of the cartilaginous skull: from a lancelet towards a human face. Seminars Cell and Dev. Biol. 91, 2–12. 10.1016/j.semcdb.2017.12.007 29248472

[B61] Kaucka M. Ivashkin E. Gyllborg D. Zikmund T. Tesarova M. Kaiser J. (2016). Analysis of neural crest-derived clones reveals novel aspects of facial development. Sci. Adv. 2, e1600060. 10.1126/sciadv.1600060 27493992 PMC4972470

[B62] Kazanskaya O. V. Severtzova E. A. Barth K. A. Ermakova G. V. Lukyanov S. A. Benyumov A. O. (1997). Anf: a novel class of vertebrate homeobox genes expressed at the anterior end of the main embryonic axis. Gene 200, 25–34. 10.1016/S0378-1119(97)00326-0 9373136

[B63] Kenyon E. J. McEwen G. K. Callaway H. Elgar G. (2011). Functional analysis of conserved non-coding regions around the short stature hox gene (shox) in whole zebrafish embryos. PLoS ONE 6, e21498. 10.1371/journal.pone.0021498 21731768 PMC3123344

[B64] Korotkova D. D. Lyubetsky V. A. Ivanova A. S. Rubanov L. I. Seliverstov A. V. Zverkov O. A. (2019). Bioinformatics screening of genes specific for well-regenerating vertebrates reveals c-answer, a regulator of brain development and regeneration. Cell Rep. 29, 1027–1040. 10.1016/j.celrep.2019.09.038 31644900 PMC6871517

[B65] Kuraku S. (2013). Impact of asymmetric gene repertoire between cyclostomes and gnathostomes. Seminars Cell Dev. Biol. 24 (2), 119–127. 10.1016/j.semcdb.2012.12.009 23291292

[B66] Kuraku S. Kuratani S. (2006). Time scale for cyclostome evolution inferred with a phylogenetic diagnosis of hagfish and lamprey cDNA sequences. Zool. Sci. 23 (12), 1053–1064. 10.2108/zsj.23.1053 17261918

[B67] Kuratani S. (2005). Craniofacial development and the evolution of the vertebrates: the old problems on a new background. Zoological Sci. 22, 1–19. 10.2108/zsj.22.1 15684579

[B68] Kuzmin E. Taylor J. S. Boone C. (2022). Retention of duplicated genes in evolution. Trends Genet. 38 (1), 59–72. 10.1016/j.tig.2021.06.016 34294428 PMC8678172

[B69] Larouche O. Zelditch M. L. Cloutier R. (2019). A critical appraisal of appendage disparity and homology in fishes. Fish. Fish. 20 (6), 1138–1175. 10.1111/faf.12402

[B70] Lauder G. V. Nauen J. C. Drucker E. G. (2002). Experimental hydrodynamics and evolution: function of median fins in ray-finned fishes. Front. Cell Dev. Biol. 42 (5), 1009–1017. 10.1093/icb/42.5.1009 21680382

[B71] Laureano A. S. Flaherty K. Hinman A. M. Jadali A. Nakamura T. Higashijima S. I. (2022). Shox2 is required for vestibular statoacoustic neuron development. Biol. Open 11, bio059599. 10.1242/bio.059599 36594417 PMC9838637

[B72] Leite-Castro J. Beviano V. Rodrigues P. N. Freitas R. (2016). HoxA genes and the fin-to-limb transition in vertebrates. J. Dev. Biol. 4 (1), 10. 10.3390/jdb4010010 29615578 PMC5831813

[B73] Long H. K. Prescott S. L. Wysocka J. (2016). Ever-changing landscapes: transcriptional enhancers in development and evolution. Cell 167, 1170–1187. 10.1016/j.cell.2016.09.018 27863239 PMC5123704

[B115] Marinić M. Aktas T. Ruf S. Spitz F. (2013). An integrated holo-enhancer unit defines tissue and gene specificity of the Fgf8 regulatory landscape. Dev. Cell 24, 530–542. 10.1016/j.devcel.2013.01.025 23453598

[B74] Marlétaz F. Timoshevskaya N. Timoshevskiy V. A. Parey E. Simakov O. Gavriouchkina D. (2024). The hagfish genome and the evolution of vertebrates. Nature 627, 811–820. 10.1038/s41586-024-07070-3 38262590 PMC10972751

[B75] Nakatani Y. Shingate P. Ravi V. Pillai N. E. Prasad A. McLysaght A. (2021). Reconstruction of proto-vertebrate, proto-cyclostome and proto-gnathostome genomes provides new insights into early vertebrate evolution. Nat. Commun. 12 (1), 4489. 10.1038/s41467-021-24573-z 34301952 PMC8302630

[B76] NCBI Resource Coordinators (2025). NCBI BLAST. Bethesda (MD): National Center for Biotechnology Information.

[B77] Ohno S. (1970). Evolution by gene duplication. Berlin, Heidelberg: Springer. 798812.

[B78] Onimaru K. Kuraku S. (2018). Inference of the ancestral vertebrate phenotype through vestiges of the whole-genome duplications. Briefings Funct. Genomics 17 (5), 352–361. 10.1093/bfgp/ely008 29566222 PMC6158797

[B79] Prummel K. D. Nieuwenhuize S. Mosimann C. (2020). The lateral plate mesoderm. Development 147 (12), dev175059. 10.1242/dev.175059 32561665 PMC7328003

[B80] Rao E. Weiss B. Fukami M. Rump A. Niesler B. Mertz A. (1997). Pseudoautosomal deletions encompassing a novel homeobox gene cause growth failure in idiopathic short stature and Turner syndrome. Nat. Genet. 16 (1), 54–63. 10.1038/ng0597-54 9140395

[B81] Rastogi S. Liberles D. A. (2005). Subfunctionalization of duplicated genes as a transition state to neofunctionalization. BMC Evol. Biol. 5, 28. 10.1186/1471-2148-5-28 15831095 PMC1112588

[B82] Redmond A. K. Casey D. Gundappa M. K. Macqueen D. J. McLysaght A. (2023). Independent rediploidization masks shared whole genome duplication in the sturgeon-paddlefish ancestor. Nat. Commun. 14 (1), 2879. 10.1038/s41467-023-38714-z 37208359 PMC10199039

[B83] Rees J. M. Sleight V. A. Clark S. J. Nakamura T. Gillis J. A. (2023). Ectodermal wnt signaling, cell fate determination, and polarity of the skate gill arch skeleton. eLife 12, e79964. 10.7554/eLife.79964 36940244 PMC10027317

[B84] Rosin J. M. Abassah-Oppong S. Cobb J. (2013). Comparative transgenic analysis of enhancers from the human SHOX and mouse Shox2 genomic regions. Hum. Mol. Genet. 22, 3063–3076. 10.1093/hmg/ddt163 23575226

[B85] Rosin J. M. Kurrasch D. M. Cobb J. (2015). Shox2 is required for the proper development of the facial motor nucleus and the establishment of the facial nerves. BMC Neurosci. 16, 39. 10.1186/s12868-015-0176-0 26156498 PMC4495855

[B86] Rubinstein M. De Souza F. S. J. (2013). Evolution of transcriptional enhancers and animal diversity. Philos. Trans. R. Soc. Lond B Biol. Sci. 368, 20130017. 10.1098/rstb.2013.0017 24218630 PMC3826491

[B87] Sabherwal N. Bangs F. Röth R. Weiss B. Jantz K. Tiecke E. (2007). Long-range conserved non-coding SHOX sequences regulate expression in developing chicken limb and are associated with short stature phenotypes in human patients. Hum. Mol. Genet. 16 (2), 210–222. 10.1093/hmg/ddl470 17200153

[B88] Sacerdot C. Louis A. Bon C. Berthelot C. Roest Crollius H. (2018). Chromosome evolution at the origin of the ancestral vertebrate genome. Genome Biol. 19 (1), 166. 10.1186/s13059-018-1559-1 30333059 PMC6193309

[B89] Sawada R. Kamei H. Hakuno F. Takahashi S. I. Shimizu T. (2015). *In vivo* loss of function study reveals the short stature homeobox-containing (shox) gene plays indispensable roles in early embryonic growth and bone formation in zebrafish. Dev. Dyn. 244 (2), 146–156. 10.1002/dvdy.24239 25483930

[B90] Seixas M. J. Domingues R. R. Antunes A. (2023). Decoding the transcriptome of sharks, rays, and chimaeras: insights into their physiology, morphology, evolution, and biomedical applications. Fishes 8 (5), 271. 10.3390/fishes8050271

[B91] Shears D. J. Vassal H. J. Goodman F. R. Palmer R. W. Reardon W. Superti-Furga A. (1998). Mutation and deletion of the pseudoautosomal gene SHOX cause leri-weill dyschondrosteosis. Nat. Genet. 19 (1), 70–73. 10.1038/ng0198-70 9590293

[B92] Shimeld S. M. Donoghue P. C. (2012). Evolutionary crossroads in developmental biology: cyclostomes (lamprey and hagfish). Development 139 (12), 2091–2099. 10.1242/dev.074716 22619386

[B93] Shubin N. Tabin C. Carroll S. (2009). Deep homology and the origins of evolutionary novelty. Nature 457, 818–823. 10.1038/nature07891 19212399

[B94] Simakov O. Marlétaz F. Yue J. X. O'Connell B. Jenkins J. Brandt A. (2020). Deeply conserved synteny resolves early events in vertebrate evolution. Nat. Ecol. and Evol. 4 (6), 820–830. 10.1038/s41559-020-1156-z 32313176 PMC7269912

[B95] Sleight V. A. Gillis J. A. (2020). Embryonic origin and serial homology of gill arches and paired fins in the skate, *leucoraja Erinacea* . eLife 9, e60635. 10.7554/eLife.60635 33198887 PMC7671686

[B96] Soldatov R. Kaucka M. Kastriti M. E. Petersen J. Chontorotzea T. Englmaier L. (2019). Spatiotemporal structure of cell fate decisions in murine neural crest. Science 364, eaas9536. 10.1126/science.aas9536 31171666

[B97] Striedter G. F. Northcutt R. G. (2019). “The origin of jaws and paired fins: the age of fishes,” in Brains through time: a natural history of vertebrates (Oxford Academic). 10.1093/oso/9780195125689.003.0003

[B116] Symmons O. Pan L. Remeseiro S. Aktas T. Klein F. Huber W. (2016). The Shh topological domain facilitates the action of remote enhancers by reducing the effects of genomic distances. Dev. Cell 39, 529–543. 10.1016/j.devcel.2016.10.015 27867070 PMC5142843

[B98] Tamura K. Stecher G. Kumar S. (2021). MEGA 11: molecular evolutionary genetics analysis version 11. Mol. Biol. Evol. 38 (7), 3022–3027. 10.1093/molbev/msab120 33892491 PMC8233496

[B99] Tanaka M. Onimaru K. (2012). Acquisition of the paired fins: a view from the sequential evolution of the lateral plate mesoderm. Evol. Dev. 14 (5), 412–420. 10.1111/j.1525-142X.2012.00561.x 22947314

[B100] Tereshina M. B. Ivanova A. S. Eroshkin F. M. Korotkova D. D. Nesterenko A. M. Bayramov A. V. (2019). Agr2‐interacting Prod1‐like protein Tfp4 from *Xenopus laevis* is necessary for early forebrain and eye development as well as for the tadpole appendage regeneration. Genesis 57, e23293. 10.1002/dvg.23293 30912273

[B101] Theveneau E. Mayor R. (2012). Neural crest delamination and migration: from epithelium-to-mesenchyme transition to collective cell migration. Dev. Biol. 366, 34–54. 10.1016/j.ydbio.2011.12.041 22261150

[B102] Thisse B. Thisse C. (2004). “Fast release clones: a high throughput expression analysis,” in ZFIN direct data submission. Available online at: http://zfin.org.

[B103] Thompson A. W. Hawkins M. B. Parey E. Wcisel D. J. Ota T. Kawasaki K. (2021). The bowfin genome illuminates the developmental evolution of ray-finned fishes. Nat. Genet. 53, 1373–1384. 10.1038/s41588-021-00914-y 34462605 PMC8423624

[B104] Wilson M. V. H. Hanke G. F. Märss T. (2007). “Paired fins of jawless vertebrates and their homologies across the “agnathan”-gnathostome transition,” in Major transitions in vertebrate evolution. Editors Anderson J. S. Sues H.-S. (Bloomington, IN: Indiana University Press), 122–149.

[B105] Woltering J. M. Irisarri I. Ericsson R. Joss J. M. P. Sordino P. Meyer A. (2020). Sarcopterygian fin ontogeny elucidates the origin of hands with digits. Sci. Adv. 6 (34), eabc3510. 10.1126/sciadv.abc3510 32875118 PMC7438105

[B106] Xu J. Zhang L. Chen X. Zhang Y. Wang Y. Fan Y. (2019). Shox2 regulates osteogenic differentiation and pattern formation during hard palate development in mice. J. Biol. Chem. 294, 18294–18305. 10.1074/jbc.RA119.008801 31649032 PMC6885637

[B107] Yano T. Tamura K. (2013). The making of differences between fins and limbs. J. Anat. 222, 100–113. 10.1111/j.1469-7580.2012.01491.x 23256837 PMC3552418

[B108] Yokokura T. Kamei H. Shibano T. Yamanaka D. Sawada-Yamaguchi R. Hakuno F. (2017). The short-stature homeobox-containing gene (shox/SHOX) is required for the regulation of cell proliferation and bone differentiation in zebrafish embryo and human mesenchymal stem cells. Front. Endocrinol. 8, 125. 10.3389/fendo.2017.00125 28642734 PMC5462919

[B109] Yu L. Gu S. Alappat S. Song Y. Yan M. Zhang X. (2005). Shox2-deficient mice exhibit a rare type of incomplete clefting of the secondary palate. Development 132, 4397–4406. 10.1242/dev.02013 16141225

[B110] Yu L. Liu H. Yan M. Yang J. Long F. Muneoka K. (2007). Shox2 is required for chondrocyte proliferation and maturation in proximal limb skeleton. Dev. Biol. 306 (2), 549–559. 10.1016/j.ydbio.2007.03.518 17481601 PMC2062576

[B111] Yu D. Ren Y. Uesaka M. Beavan A. J. S. Muffato M. Shen J. (2024). Hagfish genome elucidates vertebrate whole-genome duplication events and their evolutionary consequences. Nat. Ecol. and Evol. 8, 519–535. 10.1038/s41559-023-02299-z 38216617 PMC10927551

[B112] Zaraisky A. G. Lukyanov S. A. Vasiliev O. L. Smirnov Y. V. Belyavsky A. V. Kazanskaya O. V. (1992). A novel homeobox gene expressed in the anterior neural plate of the Xenopus embryo. Dev. Biol. 152, 373–382. 10.1016/0012-1606(92)90144-6 1353734

[B113] Zhang J. Wagh P. Guay D. Sanchez-Pulido L. Padhi B. K. Korzh V. (2010). Loss of fish Actinotrichia proteins and the fin-to-limb transition. Nature 466 (7303), 234–237. 10.1038/nature09137 20574421

[B114] Zhao W. J. Zhu M. (2010). Siluro-devonian vertebrate biostratigraphy and biogeography of China. Palaeoworld 19 (1-2), 4–26. 10.1016/j.palwor.2009.11.007

