# Supplementary material for: The subfunctionalization of shox and shox2 paralogs in shark highlights both shared and distinct developmental mechanisms of branchial arches and fins
Source: Front Cell Dev Biol. 2025 Oct 1;13:1667637. doi: 10.3389/fcell.2025.1667637 (PMC12521223; doi:10.3389/fcell.2025.1667637)
Supplement: Supplementary file 1 [file Table1.pdf]

Supplementary Table 1. Comparison of reported *shox* and *shox2* genes expression across representatives of different gnathostome classes.

	**Shox expression**	**Shox2 expression**	**Reference**
**Chondrichthyes (grey bamboo shark)**	Mandibular arch;<br>Branchial arches;<br>Paired (pectoral and pelvic) and median (dorsal, anal) fins;<br>Caudal denticles;<br>Frontonasal prominence;<br>Craniofacial ganglia	Paired fins (posterior-proximal region)<br>Craniofacial ganglia	This article
**Teleostei (*Danio rerio*)**	Mandibular and hyoid arches (ventral-intermediate domain);<br>Branchial arches;<br>Pectoral fin buds;<br>Olfactory pits;<br>Hatching gland;<br>Heart;<br>Mesencephalon;<br>Rhombencephalon;<br>Notochord	Pectoral fin bud (AER);<br>Otic placodes;<br>Heart (sinoatrial node);<br>Nervous system	Kenyon et al., 2011;<br>Sawada et al., 2014;<br>Askary et al., 2017;<br>Blaschke et al., 2007<br>Laureano et al., 2022<br>Thisse, B. and Thisse, C., ZFIN Direct Data Submission ID: ZDB-PUB-040907-1, ZDB-GENE-040426-1457
**Amphibian (axolotl, *Xenopus*)**	Limb buds	Limb buds (posterior - proximal part);<br>Sinus venosus	Duerr et al., 2025;<br>Espinoza-Lewis et al., 2009
**Aves (*Gallus gallus*)**	Limb buds (proximal 2/3);<br>Branchial arches (intermediate domain)	Limb buds (posterior-proximal part);<br>Sinus venosus	Tiecke et al., 2006;<br>Sabherwal et al., 2007
**Mammalia (*Mus musculus*)**	*Shox* gene is absent in mouse	Proximal limb (stylopodium);<br>Facial motor nucleus and nerves	Abassah-Oppong et al., 2024;<br>Gu et al., 2008;<br>Yu et al., 2005;<br>Rosin et al., 2015;<br>Xu et al., 2019
**Mammalia (*Homo sapiens*)**	Embryonic limbs;<br>1st and 2nd pharyngeal arches	Embryonic limbs (more proximally than SHOX);<br>1st, 2nd and 3rd pharyngeal arches;<br>Nasal process;<br>Pharyngeal arches;<br>Cardiac inflow tract	Clement-Jones et al., 2000
