# Supplementary material for: The subfunctionalization of shox and shox2 paralogs in shark highlights both shared and distinct developmental mechanisms of branchial arches and fins
Source: Front Cell Dev Biol. 2025 Oct 1;13:1667637. doi: 10.3389/fcell.2025.1667637 (PMC12521223; doi:10.3389/fcell.2025.1667637)

Supplementary Figure 2. Comparative expression of *shox* and *shox2* in *C. griseum* embryos at stages 28 – 30 (after Ballard et al., 1993). In contrast to *shox2*, the *shox* gene is expressed in the mandibular arch and the median (dorsal) fins. Moreover, the domain of *shox* expression in the paired fins and branchial arches is broader than that of *shox2*. Expression patterns that were reproduced in at least 80% of cases were considered reliable.

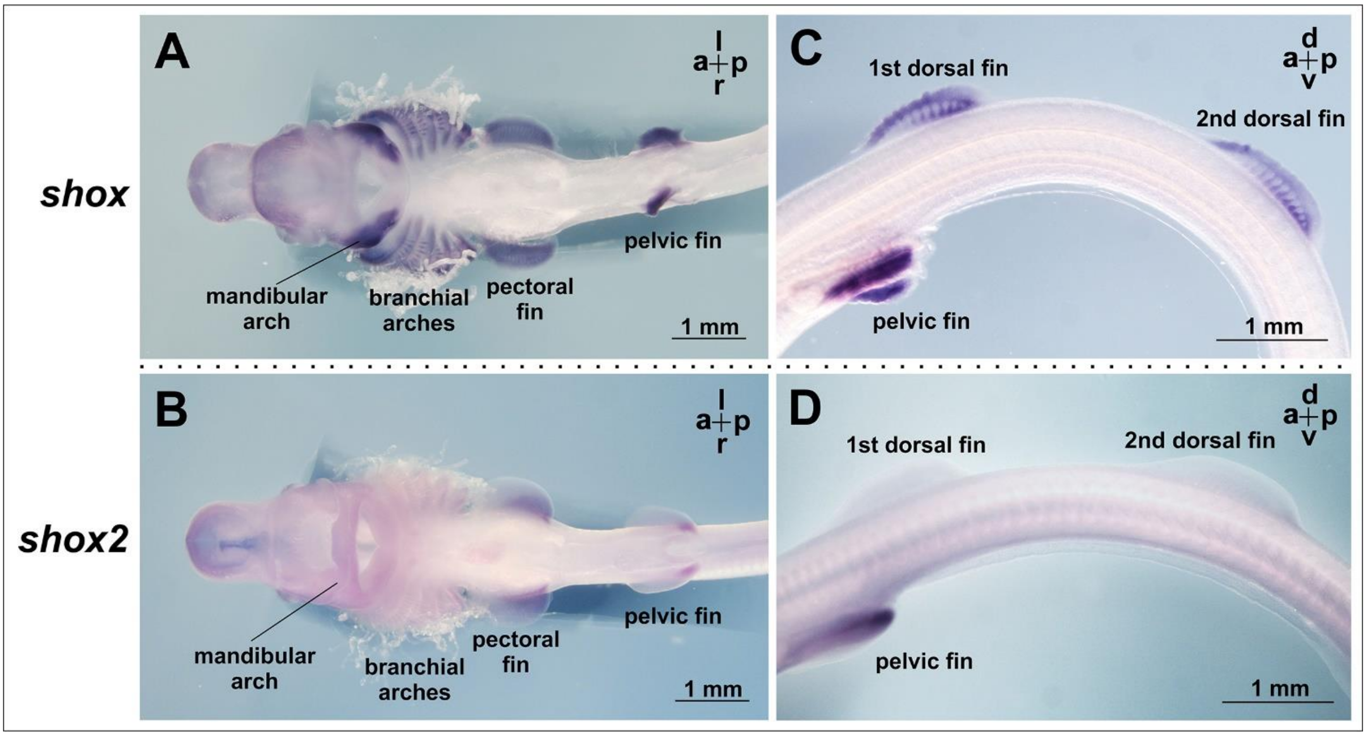

Supplement: Supplementary file 2 [file Image2.pdf]
