# Supplementary material for: The subfunctionalization of shox and shox2 paralogs in shark highlights both shared and distinct developmental mechanisms of branchial arches and fins
Source: Front Cell Dev Biol. 2025 Oct 1;13:1667637. doi: 10.3389/fcell.2025.1667637 (PMC12521223; doi:10.3389/fcell.2025.1667637)
Supplement: Supplementary file 3 [file DataSheet1.pdf]

>PaCl_Shox2(CAB4032863.1)
MEKLTAFVSRFEGEKTSSSEDIVAHSSRTSTSSPVFSSSHAFNIANILSKETGSRYRNEVRGEHLQLGSN
MPEASSGCERESHTGAIRVKSSRNRKNFSAEQLSELERLFDQTHYPDAFMREAIARRLMLSESRVQIWFQ
NRRAKSRRQEVNSNRGTGLPATKTTYSENHALQELFQRWSHRPYTFPTLNLHSPGSSLIPLTHDHREAYC
DRSTALKNSQRQSSIAELRMKARQHTETYMSNH

>RhPr_Shox2-like(XP_074000194.1)
MEELTHFVSKSFDTRLKTPSTSPSLLEPEEPASPQPLSPQNLGMGSGRNSPSANKNSTASNKQRRSRTNF
TLEQLNELERLFDETHYPDAFMREELSQRLGLSEARVQVWFQNRRAKCRKHENQMQKATGLLVSSQVASN
GTSRLEQCSRVAPYLNMPRLPFPPQTNAANFTSAFDPALITAAHQYLALSAAGLSPAAAGTSSPMLLCPQ
YGLAALAALHHDRLLTKNSSIADLRLKAKRHAEALGLNREAAGSPPPHL

>MiDe_Pmhp2(XP_053597554.1)
MEQLTQFVSKSFDHANANRESIRNNTRTANLVCSPASGTITLSVAHLIGSIDNASSTDSNVKPAPDQSPD
DSSTEERHHTASPESRLQPEDLSVATKVTKESVKQNTDTVLLPLKTPELKLSVRKLLGCSPSPPPVSRDL
SPSSPVNVQEAKVSSPQSLDSRCDKTTRKILVEDNSKGPNCRGTASTGGGGGGNGKQRRSRTNFTLEQLA
ELERLFDETHYPDAFMREELSQRLGLSEARVQVWFQNRRAKCRKHESQLHKGGMVLAPRSPPTSLEPCRV
APYLPALRLHPPSVQSSPNVSANTSAASAFDPAVLHYAAAGGLFCLPPSHPQVHPLSLAASFAAAAARSK
NSSIADLRLKARRHQEALGLDRPA

>LiPo_Shox-like(XP_013787195.1)
MEQLAEFVSKSFTTNDRSTLTASLPHPCLTTAHTFTSHLPLHPPGPRGLSTVMNRLMLPHVFHPGFPSPS
PLTTSSLVSPTVSSHSYGDDEDSGERKGSPSSARSTPSTPSERKGDRKPNITSVTSTKEAERVSDEQGDQ
SEADSCTNPSEENLKPEENKETANSKPSTHSPAHSVDTVNSHSSKSAKQRRSRTNFTLEQLNELERLFDE
THYPDAFMREELSQRLGLSEARVQVWFQNRRAKCRKHETQVQKNLLMAASTPIESSRIAPYINVSSTSPR
LPQERYLVPTTHYLPYADPAFLAAAHQYSVAAAAAAQVVHGTQPHPSFFLYPTPTHPFSLSALLAADRLN
NKNTSIADLRLKAQKHAAALGL

>LiPo_Shox2-like(XP_013784018.1)
MEQLAEFVSKSFTTGDRPTLTTSLPHPCLAPVHPFSSHLSLHPPGPRGLNSVVDRLMLPHLFPPGFPNPP
LLPPLTTTAMVSPAVSSRSCGDDEDSRNRKESPSSARSTPSSPSERKIDKKPSVSSDKEAKKTSDEHEEQ
SEDDSNPNLSEENIKPEEKQEIDNNKSNAQSPAPSVDTTNSHSSKTAKQRRSRTNFTLEQLNELERLFDE
THYPDAFMREELSQRLGLSEARVQVWFQNRRAKCRKHESQVQKNLLMAASTPIESSRIAPYINVSSTPPR
LPQERYLVPTTHYLPYADPAFLAAAHQYSVAAAAAAQVAHGTHPHPSFFFYPSPTHPFSLSALMAADRLS
NKNTSIADLRLKAQKHAAALGL

>DrPo_Shox2-like(XP_052217932.1)
MEGLTQFVSKSFENKTKVEVCDNTSETVWTAHAASRDRPSSSPSSHIDAETSIDCDVTVDDEPTDLSVSK
IDVCSSRAVTPEESIEPDESGDDSKVACSDNENDDQPKLKQRRSRTNFTLEQLNELERLFDETHYPDAFM
REELSQRLGLSEARVQVWFQNRRAKCRKQESQMQKAGIMLTSSGNMPQLDACRVSPFLNMSTIRQPLDRL
HFPPFLPYFPLTSASGLPPPLHPMLLYHHHYAAAALNYASLPVESGFKATKTTSIADLRLKARQHLASLG
LSNI

>DrPo_Shox-like(XP_052224109.1)
MDSKEVLDWGESGSDLSSKSGCNRPVSSDDSKVACSDNENDDQPKLKQRRSRTNFTLEQLNELERLFDET
HYPDAFMREELSQRLGLSEARVKVWFQNRRAKCRKQERQMQKAGIMLTCSGNMPQLDACRVSPFLNMSTI
RQPLDRRHFPPFLPYFPLTSAHGLPLHPMLLYRHHYAAVALNYASLPVESGFKATKTTSIADLRLKARQH
LASLGLSNI

>OcBi_Shox2(XP_052830813.1)
MRKMERLTEFVSKSFDAGSDQQKNDFNEKMQLISELNSPNIESSSQFDNHQSAVSKAVGVAAAAAAAILP
SPYRQSLVEVTSSTSSTGSSGHLPRESNQDMTTIDVVDEAELTSSKSVNSISSASDLNCRQSFSDCTKDN
RSDVEDDDQPKLKQRRSRTNFTLEQLNELERLFDETHYPDAFMREELSQRLGLSEARVQVWFQNRRAKCR
KQESQMQKAGLLLQSPGTPLDTCRVAPFFNMPPIREPMERFAMPPFPPYYPYHAAAAAAAASQHPLHPIF
FYPHHYTALNYAMIPDSSFKTNKSSSIADLRLKAKKHLATLGL

>HaAs_Shox2-like(XP_067670467.1)
MEGLTEFVSKSFEECTDNIKKETLSVPVVCDDKNNLDSENIKITSSGSEDEKSCDESDDELSIGGREKGV
LTVKTDLKDGKEKEEEGSPKVKQRRSRTNFTLEQLNELERLFDETHYPDAFMREELSQRLGLSEARVQVW
FQNRRAKCRKQESQMQKAGLLMQSQGTPLDPCRVAPFMNLPQVREPGIERLPYPPFYPFLPLHPAAAAAA
AAAAASASQHPIHPMLLYHHHLAALNYTALPDSSFRSAKNSSIADLRLKARQHLASLGI

>OwFu_unnamed(CAH1781372.1)
MEGLTNFVSKSFDASRLLQDAILMRETRTPDENHHTTEVAHATKQDSHRLKQASPKELPENKHSVEAILS

```
DTYKPKINTINDLSMKSRQFQFTQRDIMTTNETKETNKNHSISKTEELKIDVENDDFSDTYNEPKDMTHL
KEDSDDDTPKLKQRRSRTNFTLEQLNELERLFDETHYPDAFMREELSQRLGLSEARVQVWFQNRRAKCRK
QESQIHKGLILGSTPPSIDGCRVAPYVNMPSMRMAFDRLHLPHINPYDGSHASHPALPSMLFYPPPPYPL
NLSALADSSFKATKTSSIADLRLKAKQHTASLGLK

>CaTe_hypothetical_proteim(ELU02974.1)
MEGGHASPMMMNFSRDDVSRSPSPVVVDENSPEASPSSSRSREAMKDAPSPPRRPADELRVSPPPQPPPR
LPVAMQEAFSARPQMQPNIHADIAMEKQDERSRSPILRGPPPPLPLPRASSPRLPMMQDDSSAHDVRDGG
EDDPPKIKQRRSRTNFTLEQLNELERLFDETHYPDAFMREELSQRLALSEARIQVWFQNRRAKCRKQESQ
LQKGLLLSPTVDGCRVAPYVNMPSMRMAFDRLHMHHLRHLHDPTAALLSPHPSATAAAVAAAAAMPSMLF
YPSPHYAVNLALAQNVGSSVTAQTKNSSIADLRMKAKKHSAALGF

>AcPl_Shox2-like(XP_022105055.1)
MTLFGPGDSDLARDRLALFKRGTPHLSKPSSAKRPKSTTEKKKHGSKSPWEDVSKVSEPQQITEDAISPV
GHEDEDVDMDTDPDEDLALHEDADIAAAEQEDRAGVPSRQPREPKETVSGSPSPVQGLMASHPSKLKQRR
SRTNFTVEQLGELEKLFDETHYPDAFMREELSRKLGLSEARVQVWFQNRRAKCRKQEHLGGKGTPIGASS
NVDTCRVAPYLSMGSLRMPFDRVQEQLQLNLTTTVTAAPSVPRPLPSIFTHAPSLMMFPPPAYAFPLATL
MSSMIRPGGGSKTSSIADLRMKARQHAAALGLHSFLSQ

>ApJa_uncharacterised(>XP_071838769.1)
MEELEAFVSKSFAGQDLTPTEPSEEQKRAAVSIRKCTEWLSLIQGSANSTTANAKKGKVASTNPKSKMRP
ETDRTEKETLDGAAKPEEETQEDTGNKERKRTIESDNEEPCVVKRQKSDGESNNEVNIGGNKNAERLAER
GGRSTEKYPALDVSNISTGKSKTSQSEVQDESHMTQKEQNNNEITPTRLKESAETGPIEQVAIVTAHPQR
PRLWNPCHLSPDSTGAREGTVVDEDQGPLDMSGADGINLGHHGKLKQRRSRTNFTVEQLGELEKLFDETH
YPDAFMREELSKKLGLSEARVQVWFQNRRAKCRKQENQSSKGSGVGPSTSVDTCRVAPYLSMGAMRMPFE
RVQEQLQLNLSPPCKTPMESSQGRLPPVPPLPTSLLAHAHPFMFLPAPSPSYALQMATLVGSLMARGPGG
PMGLGMAKNSSIAELRMKARQHAAALGLQPFLSPMDH

>HoLe_Shox(KAJ8031104.1)
MEELEAFVSKSFAGQDMAPTEPNEEQKRAAASIRKCTEWLSLIQGTPTGPSSAIKKMKPGSSPKNKTRSE
TVRHDKDPLEAEEAEVNESASQNRKRTGATEGEDEPLTKRLRTEDDVLGTESRQDDHVPTKLGNETGSFS
RLHCTQETDHPKNDERNPGKQKEIADSQEEPNNNDVNASQMNQPKETSLTQQEKVTASQPKPRLWNPCHL
SPDSTAAEEDANDDQGALSMTGGDSNPLAHHGKLKQRRSRTNFTVEQLGELEKLFDETHYPDAFMREELS
KKLGLSEARVQVWFQNRRAKCRKQENQSSKGSGVGASTSVDTCRVAPYLSMGAMRMPFERVQEQLQLNMS
PSCKSPMESTPSRIPPPPIPASLLAHTHPFMFLPAPSPSYALQMATLVGSLMSRAPGPMGLGMTKNSSIA
ELRLKARQHAAALGLQPFLSPLDH

>PtFl_Shox-like(XP_070538286.1)
MREELSNRLGLSEARVQVWFQNRRAKCRKQENQIQKGILLNAGVDTCRVAPYVNMGAVRMPFERVQAHLH
LNGTTTSSAMVTPTNATTFIAPSHSMVMFPHPGFPVAMTTLLDTAKSAKSSSIADLRLKARKHAAALGLR
HLAL

>BrFl_Shox2-like(XP_035693605.1)
MEELTAFVSKSFDGKDKAEEIREERTYKEVLGDLKGDMDQRHGDSGAESDVDGDDLCPVGSPGLPVGLGG
GENSPGPENKDPSKPGEKKDDIESPNSKDGKLKQRRSRTNFTLEQLQELERLFDETHYPDAFMREELSQR
LGLSEARVQVWFQNRRAKCRKQENQLQKGGDMLCSALRGNLTTRVSAHTGMMMGSNLDTCRVAPYMNMGT
VRMPFERVQAHLQFDAAAHAPHPSMMLYAPAPYALPITSMAGSLAAGSLEVTKNSKNNSIADLRLKARKH
AEALGL

>OiDi_unnamed(CBY23553.1)
MEELAKFVSGQFSAPKHSNERCFKDVLQTQESSSPSHEEADQPQRIRLFRPHEHVPANALDLSPTSIHHQ
DAQISPDLSKDKQKQRRSRTNFTMEQIHALESLFEQTHYPDAFMREELSQNLGLSEARVQVWFQNRRAKS
RKQESCEATSASLPGTLRQHARMTGFSMRSSSALHCRPQIAVQPPIQLCDELVSRSLFSAYHQANDSAQF
ARISALLQLQRLRQPALISPLLRFPPLFHIPPVQLPCTLPDNQAQANVTDADAVKDEKDENEDNKDLDVV

>PeMa_Shox-like(XP_032813768.1)
MEELARFVRASFAGGGRRPRSSGARHGGGETQAEAPSGDSNPWGGGGSALRRSRRCRTSFSAEQLQQLER
AFEESHYPDGGARLALSARLHLPETRVQVWFQNRRAKSRKQEMQARRGVMLRPGLDGHRVAPYIHMGVTR
LHHKQVPAHLCCDHVTRRRPQSPGTELGAGAGVEPATFPSWGLPPVPFALALPLGLEAASDGAQVSSVSK
HSSLADLRMKARRHAASLGL

>PeMa_Shox-like(XP_032836218.1)
MEELRAFVSKSFERRYRPQDGGVGVVGATFRDVLRAGSPRCGGAGGPPQGDLASPRGPSPPSSPAPSSPS
SPTSSTSSSLPRRRPKQRRSRTNFSAEQLGELEGLFDETHYPDAFMREELSQRLGLSEARVQVWFQNRRA
```

KCRKQENQLHRGVFFSASSHLDACHVAPYLSINSLRLPFQQVQMEGISTHATLQGTHPPYVLFPPPPPPP
PPPPPPHSHPHLHHHHHHHHPHHHQHHHHQQHEQESQTVDEVDEVDEAEARGGPRAGRELNVNLASD

>PeMa_Shox2-like(XP_032827813.1)
MEELTAFVSKSFEGKVKDRKDGITYREVLESGSLRARSGMATDEIVATAAAAAAAAAAREEAMAGSLSAG
LAPRLPMLPRPPSSKELGLDGDSEREDSDFGVSKGRDDSKERREESRAEEEEGQAKLKQRRSRTNFTLEQ
LNELERLFDETHYPDAFMREELSQRLGLSEARVQVWFQNRRAKCRKQENQMHKVFTACRPGVLIGTTSHL
EACRVAPYVNMGALRMPFQQVQAQLQLEGVAAHAHHHLHSHLAAHAPYMMFPPPPFGLPSLASIADSASA
VAAAAAAAKTSTKNSSIADLRLKARKHAAALGL

>CaMi_Shox(XP_042190331.1)
MEELTAFVSKSFDQKSKDKKEAITYREVLESGLARSRELSGSESSCQDITENSHCPPHSYRDPEPENRTL
REPNPGRTSEGIYDCKEKREEVKSEDEDGQTKLKQRRSRTNFTLEQLNELERLFDETHYPDAFMREELSQ
RLGLSEARVQVWFQNRRAKCRKQENQMHKGVILGSGSHMDACRVAPYVNMGALRMPFQQVQAQLQLDGVA
HAHPHLHHHLAAHAPYLMFPPPPFGLPIASIAESASAAAAAAAAAAVKTSSKNSSIADLRLKARKHAEAL
GL

>CaMi_Shox2(XP_042195080.1)
MEELTAFVSKSFDQKVKEKKEGITYREVLESGPVRAREPCMGEGNRDEVNGAQRGSSRCPSTEPETGPER
PGDSGTPKLADPGNGDIKERKEDAKPMEEETQTKIKQRRSRTNFTLEQLNELERLFDETHYPDAFMREEL
SQRLGLSEARVQVWFQNRRAKCRKQENQLHKGVLIGAANQFEACRVAPYVNVGTLRMPFQQDVHSNMPPL
SFQVQAQLQLDSVAHAHHHLHPHLAAHAPYMMFPPPHFGLPLATLAESATVVAAAAAAKTTSKNSSIADL
RLKAKKHAAALGL

>ChPl_Shox(XP_043548272.1)
MEELTAFVSKSFEQKNKDKKEAITYREVLESGLARSRELSGSDSSCQEITDTSHCTTHVYKDVDNENEKV
KDLNSARTSEGIYECKEKREDVKSEDEDGQTKLKQRRSRTNFTLEQLNELERLFDETHYPDAFMREELSQ
RLGLSEARVQVWFQNRRAKCRKQENQMHKGVILGTGSHLDACRVAPYVNMGALRMPFQQVQAQLQLDGVA
HAHSHLHHHLAAHAPYLMFPPPPFGLQIASIAESASAAAAAAAAAAVKTSSKNSSIADLRLKARKHAEAL
GL

>ChPl_Shox2(XP_043558048.1)
MEELTAFVSKSFDQKVKEKKDGITYREVLESGPVRAREPSLSEGSRDEMISAQRGASRSPSSEPEIGPER
PRDSGTPKLTDPGLKRYHVAMEKATHNSTTSALEKTEGSGDIKDRKEDPKPVEEEAQTKIKQRRSRTNFT
LEQLNELERLFDETHYPDAFMREELSQRLGLSEARVQVWFQNRRAKCRKQENQLHKGVLIGTTSQFEACR
VAPYVNVGALRMPFQQENHCNMTPLSFQVQAQLQLDSVAHAHHHLHPHLAAHAPYMMFPPPPFGLPLATL
AESATVVAAAAAAKTTSKNSSIADLRLKAKKHAAALGL

>AcRu_Shox-like(XP_033867376.2)
MEELTAFVSKSFDQKTKESSKKESITYREVLESGLARARELGNSETNLQDISETSNHCPVHLFKEHVELE
KEKMKEFNVSRASEGIYECKEKKEEVKSEDEDGQSKLKQRRSRTNFTLEQLNELERLFDETHYPDAFMRE
ELSQRLGLSEARVQVWFQNRRAKCRKQENQMHKGVILGPANHLDACRVAPYVNMGALRMPFQQVQAQLQL
EGVAHAHHHLHPHLAAHAPYLMFPPPPFGLPITSLTESASAAAAVAAAAKSNSKNSSIADLRLKARKHAE
ALGL

>AcRu_Shox-like(XP_033866010.1)
MEELTAFVSKSFDQKTKESSKKESITYREVLESGLARARELGNSETNLQDISETSNHCPVHLFKEHVELE
KEKMKEFNVSRASEGIYECKEKKEEVKSEDEDGQSKLKQRRSRTNFTLEQLNELERLFDETHYPDAFMRE
ELSQRLGLSEARVQVWFQNRRAKCRKQENQMHKGVILGPASHLDACRVAPYVNMGALRMPFQQVQAQLQL
EGVAHAHHHLHPHLAAHAPYLMFPPPPFGLPITSLAESASAAAAVAAAAKSNSKNSSIADLRLKARKHAE
ALGL

>AcRu_Shox2-like(XP_033884420.1)
MEELTAFVSKSFDQKVKEKKEVITYREVLETGPVRGREPNSSEPSREEVNAIARGGARSPSREIDMLGPE
RTRDSCSPKIIDGNTDMKERKEDSKSMDDEAQTKIKQRRSRTNFTLEQLNELERLFDETHYPDAFMREEL
SQRLGLSEARVQVWFQNRRAKCRKQENQLHKGVLIGAASQYEACRVAPYVNVGALRMPFQQDSHCNVPPF
SFQVQAQLQLDSAVAHAHHHLHSHLAAHAPYMMFPAPPFGLPLATLAAESATAASVVAAAAAAKTTNKNS
SIADLRLKAKKHAAALGL

>AcRu_Shox2-like(XP_033894690.1)
MEELTAFVSKSFDQKVKEKKEVITYREVLETGPVRGREPNSSEPSREEMNAIARGGARSPSREIDMLGPE
RTRDSCSPKIIDGNTDMKERKEDSKAMDDEAQTKIKQRRSRTNFTLEQLNELERLFDETHYPDAFMREEL
SQRLGLSEARVQVWFQNRRAKCRKQENQLHKGVLIGAASQYEACRVAPYVNVGALRMPFQQDSHCNVPPF
SFQVQAQLQLDSAVAHAHHHLHSHLAAHAPYMMFPAPPFGLPLATLAAESATAASVVAAAAAAKTTNKNS

SIADLRLKAKKHAAALGL

>LeOc_Shox(XP_006638959.1)
MEELTAFVSKSFDQKAKESNKKESITYREVLESGLARARELGNSETNLQDITETSNHCPVHLYKDHVELE
KEKLKEFNVTRASEGIYECKEKKEEVKSEDEDGQSKLKQRRSRTNFTLEQLNELERLFDETHYPDAFMRE
ELSQRLGLSEARVQVWFQNRRAKCRKQENQMHKGVILGTASHLDACRVAPYVNMGALRMPFQQVQAQLQL
EGVTHTHPHLHPHLAAHAPYLMFPPPPFGLPIASLADSASAAAAVAAAAKSNSKNSSIADLRLKARKHAE
ALGL

>LeOc_Shox2(XP_006637636.1)
MEELTAFVSKSFDQKVKEKKEVITYREVLETGPARGREPISTEPSREEVSALARGGARSPGRETDMLGPE
RTRDTGSPKLIDGNTDMKERKEDSKPMEDETQTKIKQRRSRTNFTLEQLNELERLFDETHYPDAFMREEL
SQRLGLSEARVQVWFQNRRAKCRKQENQLHKGVLIGAASQFEACRVAPYVNVGALRMPFQQDSHCNVPPL
SFQVQAQLQLDSAVAHAHHHLHSHLAAHAPYMMFPAPPFGLPLATLAAESASAASVVAAAAAAKSTNKNS
SIADLRLKAKKHAAALGL

>DaRe_Shox(NP_001119883.1)
MEELTAFVSKSFDQKTKESSKESITYREVLESGLARARELGNSETNLQEITETNNNHCPVHLYKEHVELE
KEKLKEFSVTRASDGIYDCKDKKEDVKSEDEDAQSKLKQRRSRTNFTLEQLNELERLFDETHYPDAFMRE
ELSQRLGLSEARVQVWFQNRRAKCRKQENQMHKGVILGTASHLDACRVAPYVNMGALRMPFQQVQAQLQL
EGVGTHSHPHLHPHLAAHAPYLMFPPPPFGLPIASLADSASAAAAVVAAAKSNSKNSSIADLRLKARKHA
EALGL

>DaRe_Shox2(NP_957490.1)
MEELTAFVSKSFDQKVKEKKEVITYREVLETGSVRNRESLSADPNREEISSITRSGVRSSPVREADMLAS
ERSRDSSSPKLTDGNTDMKERKEDCKPLEDETQTKIKQRRSRTNFTLEQLNELERLFDETHYPDAFMREE
LSQRLGLSEARVQVWFQNRRAKCRKQENQLHKGVLIGAASQFEACRVAPYVNVGALRMPFQQDSHCNVPP
FSFQVQAQLQLDSAVAHAHHHLHSHLAAHAPYMMFPAPPFGLPLATLAAESASAASVVAAAAAAKTTNKN
SSIADLRLKAKKHAAALGL

>PoSe_Shox(XP_039600684.1)
MEELTAFVSKSFDQKAKESSKKESITYREVLESGLARTRDLANSDSSLQDISETGNHCPVHLFKDHAELE
KDKLKEFNMTRTSEGIYECKEKKEEVKSEDEDGQSKLKQRRSRTNFTLEQLNELERLFDETHYPDAFMRE
ELSQRLGLSEARVQVWFQNRRAKCRKQENQMHKGVILGTTNHLDACRVAPYVNMGALRMPFQQVQAQLQL
EGVAHTHPHLHPHLAAHAPYLMFPPPPFGLPIASLADTASAAAVVAAAAKSNSKNSSIADLRLKARKHAE
ALGL

>PoSe_Shox2(XP_039612220.1)
MEELTAFVSKSFDQKVKEKKEIITYREVLESGPVRVRDPSASEPSREEVNAFTRGSARSPSRETDILGPE
RTRDAGSPKLIDGATDMKERKEDSKPMEDETQTKIKQRRSRTNFTLEQLNELERLFDETHYPDAFMREEL
SQRLGLSEARVQVWFQNRRAKCRKQENQLHKGVLIGAASQFEACRVAPYVNVGALRMPFQQDSHCNVPPL
SFQVQAQLQLDSAVAHAHHHLHSHLAAHAPYMMFPAPPFGLPLATLAAESASAASVVAAAAAAKNTNKNS
SIADLRLKAKKHAAALGL

>PrAn_Shox(XP_043928416.1)
MEELTAFVSKSFDPKSKECSSNSKKESITYREVLESGLARARELVSSEASLQELAEAASGGNSLHCPVHH
MYKEQAGESDKEKLKEFGGSLGRTTEGIYDCKEKREDVKSEDEDGQTKLKQRRSRTNFTLEQLNELERLF
DETHYPDAFMREELSQRLGLSEARVQVWFQNRRAKCRKQENQMHKVGVILGTANHLDACRVAPYVNMGAL
RMPFQQVQAQLQLEGVAHTHPHLHPHLAAHAPYLMFPPPPFGLPIASLAETASAAAVVAAAAKSNSKNSS
IADLRLKARKHAEALGL

>PrAn_Shox2(XP_043926414.1)
MSDCIHTRKRPVAVLVVAWYGKNSLKAVMEELTAFVSKSFDPKVKEKKDGITYREVLESGPTRGRELGTG
EPSRDELGATARVGARSPGRESEAGAERTRDSGTPKLTDVTMDLKERKDDCKVTDDDGQTKIKQRRSRTN
FTLEQLNELERLFDETHYPDAFMREELSQRLGLSEARVQVWFQNRRAKCRKQENQLHKGVLIGAGSQFEA
CRVAPYVNVGALRMPFQQDSYCNVPPLSFQVQAQLQLDSAVAHAHHHLHPHLAAHAPYMMFPAPPFGLPL
ATLAAESASAASVVAAAAAAKTSSKNSSIADLRLKAKKHAAALGL

>LaCh_Shox(XP_005997264.1)
MEELTAFVSKSFDQKNKESSSKKETITYREVLESGLARSRELGNSETNLQDITETSNHCPVHLYKEHVDS
EKDKLKDFNTTRASEGIYECKEKREDLKSEDEDGQTKLKQRRSRTNFTLEQLNELERLFDETHYPDAFMR
EELSQRLGLSEARVQVWFQNRRAKCRKQENQMHKGVILGTASHLDACRVAPYVNMGALRMPFQQVQAQLQ
LEGVTHAHPHLHPHLAAHAPYLMFPPPPFGLPIASLAESASAAAAVAAAAKSNSKNSSIADLRLKARKHA
EALGL

>LaCh_Shox2(XP_006002215.1)
MEELTAFVSKSFDQKVKEKKEVFTYKEVLESGPVRGRDSGSSEQSREEMNTVARGGARSPGRESDIGTER
PRDPGTPKLTDVTTELRERKDDCKTMEDETQTKIKQRRSRTNFTLEQLNELERLFDETHYPDAFMREELS
QRLGLSEARVQVWFQNRRAKCRKQENQLHKGVLIGAASQFEACRVAPYVNVGALRMPFQQDSHCNVPPLS
FQVQAQLQLDSAVAHAHHHLHPHLAAHAPYMMFPAPPFGLPLATLAAESASAASMAAVAAAAKTTNKNSS
IADLRLKAKKHAAALGL

>XeTr_Shox(XP_004911874.1)
MQFILGVKKKNNKKLPRDVSPSQCKAQGSLWIGTVRFCWAAMEELTAFVSKSFDQKSKESNGGGSSKKET
ITYREVLETGLARVRELGNSETNLQDITDTSNHCPLHLYKEHGADSDKDKLKDYGSTRVSEGIYECKEKR
DDVKSEDEDGQTKLKQRRSRTNFTLEQLNELERLFDETHYPDAFMREELSQRLGLSEARVQVWFQNRRAK
CRKQENQMHKGGYGVILGTGSHLETCRVAPYVNMGALRMPFQQVQAQLQLEGVAHAHPHLHPHLAAHAPY
LMFPPPPFGLPIASLADTASAAAVVAAAAKSNSKNSSIADLRLKARKHAEALGL

>XeTr_Shox2(XP_012818036.1)
MEELTAFVSKSFDQKIKEKKEMITYREVLESGPARGKEPGCGEGAREDGLAGNRCIGGGGGGGGGGGGAR
SPVLELDLSVERIRESGSPKLTEVSPEIKERKEELKQQALEEEGQTKIKQRRSRTNFTLEQLNELERLFD
ETHYPDAFMREELSQRLGLSEARVQVWFQNRRAKCRKQENQLHKGVLIGAGSQFEACRVAPYVNVGALRM
PFQQVQAQLQLDSAVAHAHHHLHPHLTAHAPYMMFPAPPFGLPLATLAAETATAASVVAAAAAAKTSSKN
SSIADLRLKAKKHAAALGL

>GaGa_Shox(NP_001384174.1)
MEELTAFVSKSFDQKSKESGGGGGGGNKETITYREVLESGLARSRELGNSDSALPDMTEGSNHCPVHLFK
DHVESDKDKLKEFAAGRTAEGIYECKEKREDVKSEDEDGQTKLKQRRSRTNFTLEQLNELERLFDETHYP
DAFMREELSQRLGLSEARVQVWFQNRRAKCRKQENQMHKGVILGTASHLDACRVAPYVNMGALRMPFQQV
QAQLQLEGVAHAHPHLHPHLAAHAPYLMFPPPPFGLPIASLAESASAAAVVAAAAKSNSKNSSIADLRLK
ARKHAEALGL

>GaGa_Shox(XP_015147272.2)
MEELTAFVSKSFDPKAKEKKELITYREVLESGPLRGAREPGGAAAEPGRDETGSPAGRAGGGRSPPREPD
AAAADRAADAATPKLSDVSPELKERKEDAKGMEDEGQTKIKQRRSRTNFTLEQLNELERLFDETHYPDAF
MREELSQRLGLSEARVQVWFQNRRAKCRKQENQLHKGVLIGAASQFEACRVAPYVNVGALRMPFQQVQAQ
LQLDSAVAHAHHHLHPHLAHAPYMMFPAPPFGLPLATLAESASAASVVAAAAAAKTTSKNSSIADLRLKA
KKHAAALGL

>HoSa_SHOXa(NP_000442.1)
MEELTAFVSKSFDQKSKDGNGGGGGGGGKKDSITYREVLESGLARSRELGTSDSSLQDITEGGGHCPVHL
FKDHVDNDKEKLKEFGTARVAEGIYECKEKREDVKSEDEDGQTKLKQRRSRTNFTLEQLNELERLFDETH
YPDAFMREELSQRLGLSEARVQVWFQNRRAKCRKQENQMHKGVILGTANHLDACRVAPYVNMGALRMPFQ
QVQAQLQLEGVAHAHPHLHPHLAAHAPYLMFPPPPFGLPIASLAESASAAAVVAAAAKSNSKNSSIADLR
LKARKHAEALGL

>HoSa_SHOX2a(NP_006875.2)
MEELTAFVSKSFDQKVKEKKEAITYREVLESGPLRGAKEPTGCTEAGRDDRSSPAVRAAGGGGGGGGGGG
GGGGGGGVGGGGAGGGAGGGRSPVRELDMGAAERSREPGSPRLTEVSPELKDRKEDAKGMEDEGQTKIKQ
RRSRTNFTLEQLNELERLFDETHYPDAFMREELSQRLGLSEARVQVWFQNRRAKCRKQENQLHKGVLIGA
ASQFEACRVAPYVNVGALRMPFQQDSHCNVTPLSFQVQAQLQLDSAVAHAHHHLHPHLAAHAPYMMFPAP
PFGLPLATLAADSASAASVVAAAAAAKTTSKNSSIADLRLKAKKHAAALGL

>MuMu_Shox2(NP_038693.1)
MEELTAFVSKSFDQKVKEKKEAITYREVLESGPLRGAKEPGCVEPGRDDRSSPAVRAAGGGGAGGGGGG
GGGGGGGAGGGGAGGGAGGGRSPVRELDMGAAERSREPGSPRLTEVSPELKDRKDDAKGMEDEGQTKIKQ
RRSRTNFTLEQLNELERLFDETHYPDAFMREELSQRLGLSEARVQVWFQNRRAKCRKQENQLHKGVLIGA
ASQFEACRVAPYVNVGALRMPFQQDSHCNVTPLSFQVQAQLQLDSAVAHAHHHLHPHLAAHAPYMMFPAP
PFGLPLATLAADSASAASVVAAAAAAKTTSKNSSIADLRLKAKKHAAALGL
