# Supplementary material for: The subfunctionalization of shox and shox2 paralogs in shark highlights both shared and distinct developmental mechanisms of branchial arches and fins
Source: Front Cell Dev Biol. 2025 Oct 1;13:1667637. doi: 10.3389/fcell.2025.1667637 (PMC12521223; doi:10.3389/fcell.2025.1667637)

Supplementary Figure 1. ML phylogenetic trees of Shox and Shox2 proteins of gnathostomes. Bootstraps >50 are shown. The Shox and Shox2 proteins of gnathostomes clearly segregate into two distinct clusters.

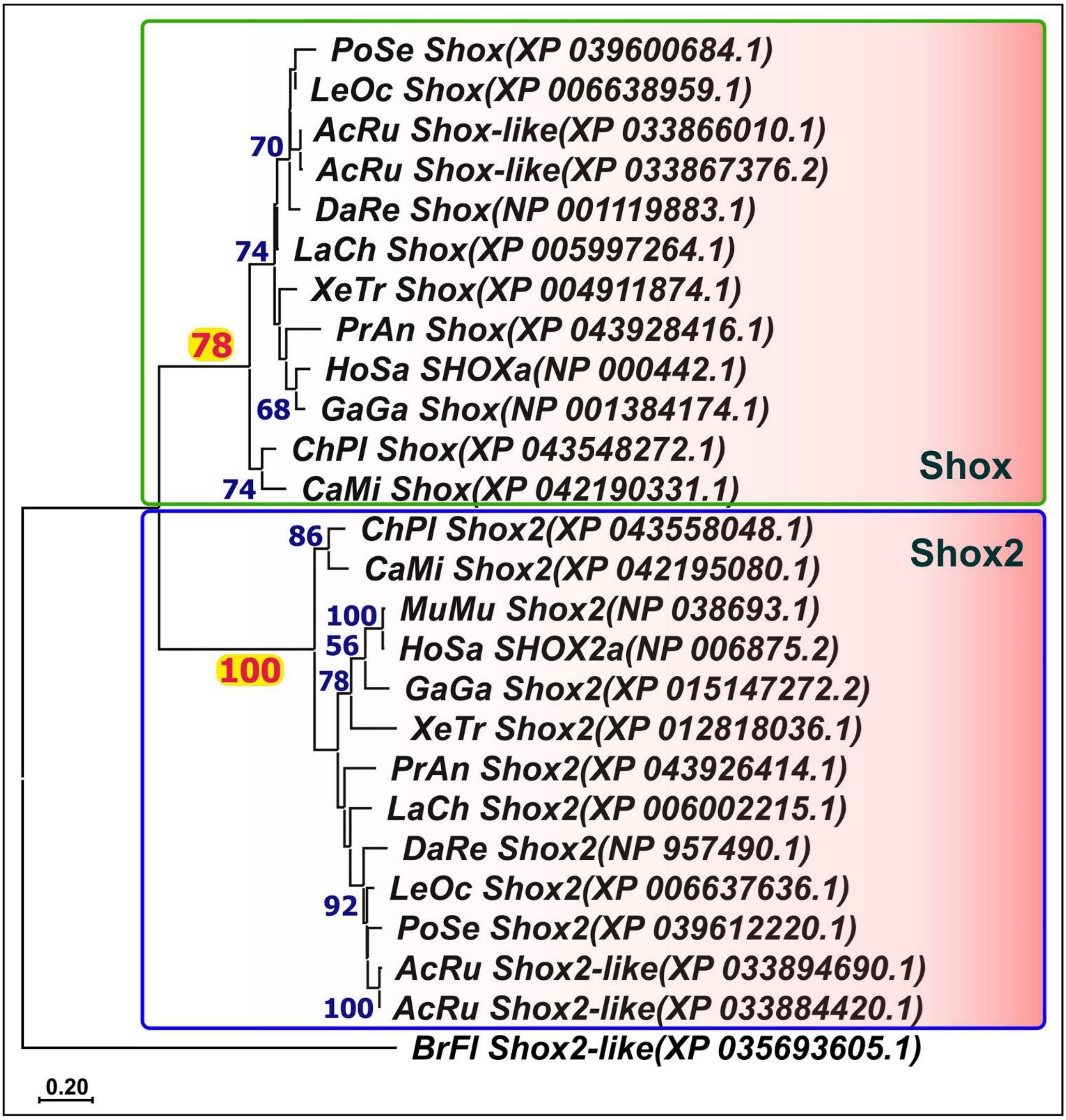

Supplement: Supplementary file 4 [file Image1.pdf]
